# Influence of Nanoparticles on Morpho-Physiological, Growth and Yield Traits of Rice (*Oryza sativa* L.) Cultivars Under Early Seedling Cold Stress at Different Developmental Stages

**DOI:** 10.3390/plants15172595

**Published:** 2026-08-25

**Authors:** Yinghui Li, Shafi Ullah, Atika Khan, Can Tan, Md. Atik Mas-ud, Muhammad Waqas, Liwu Sui, Dongliang Xiong, Jianliang Huang

**Affiliations:** Ministry of Agriculture Key Laboratory of Crop Ecophysiology and Farming System in the Middle Reaches of the Yangtze River, College of Plant Science and Technology, Huazhong Agricultural University, Wuhan 430070, China

**Keywords:** cold stress, nanoparticles, antioxidant, oxidative stress, grain yield, rice cultivars

## Abstract

Cold stress (CS) severely limits the growth and productivity of rice (*Oryza sativa* L.), particularly in temperate regions where abrupt temperature declines frequently occur during early developmental stages. In recent years, nanoparticle (NP) application has emerged as a promising approach for alleviating CS; however, systematic comparisons of different NPs across multiple growth stages remain unclear. This study evaluated the effectiveness and physiological mechanisms of four NPs (Fe_2_O_3_, ZnO, TiO_2_, and CeO_2_) in enhancing CS tolerance of rice seedlings using four cultivars with contrasting cold tolerance: two conventional cultivars (ZJZ-17, cold-sensitive; XZX-6, cold-tolerant) and two hybrid cultivars (LLY-7108, cold-tolerant; LLY-32, cold-sensitive). Seedlings were subjected to CS (14 °C day/10 °C night) for 5 days at three developmental stages (14, 21, and 28 days after emergence), followed by a 7-day recovery period under optimal conditions. CS markedly reduced plant height (34.8%), fresh weight (57.2%), dry weight (50.0%), and chlorophyll a and b contents (48%) following recovery. Foliar application of NPs significantly mitigated the adverse effects of CS, with Fe_2_O_3_ and ZnO showing the highest effectiveness. Fe_2_O_3_ treatment increased plant height, fresh weight, and dry weight by 25.6%, 43.5%, and 40.6%, respectively, relative to cold-stressed plants, while chlorophyll a and b contents increased by 41.6% and 42.2%. NPs application alleviated oxidative damage by reducing reactive oxygen species (up to 67.4%), malondialdehyde (up to 51.2%), and proline accumulation (up to 60.4%). Enhanced antioxidant defense was evidenced by increased activities of superoxide dismutase (66.6%), peroxidase (59.6%), and catalase (34.3%) under Fe_2_O_3_ treatment. Yield-related traits also showed significant recovery, with Fe_2_O_3_ increasing tiller number, spikelets per panicle, and grain yield per plant. The hybrid cultivar LLY-7108 consistently exhibited greater CS tolerance than conventional cultivars, while the cold-sensitive cultivar ZJZ-17 showed the greatest susceptibility. CS imposed at later growth stages (28-day-old seedlings) caused less damage and allowed greater recovery than early-stage stress (14-day-old seedlings). Overall, NP-mediated enhancement of photosynthesis and antioxidant capacity significantly improves CS tolerance and yield performance in rice, with Fe_2_O_3_ NPs emerging as a promising strategy for mitigating CS. These findings provide practical insights for rice cultivation in regions prone to chilling events and contribute to the development of nanoparticle-based approaches for rice production under climate stress.

## 1. Introduction

Rice (*Oryza sativa* L.) is a staple food crop and ranks one of the most widely cultivated crops globally [1]. Global rice consumption continues to rise due to population growth and increasing per capita consumption [2]. However, cold stress remains a major constraint to improving rice production in the mountainous regions of the tropics and temperate rice-growing areas [3,4]. Globally, approximately 15 million hectares of rice-growing land in 24 countries are at risk of cold-induced damage. [5]. Rice exposed to cold stress (CS) exhibits symptoms including leaf yellowing, delayed seedling growth, stunted development, wilting, reduced tillering, and ultimately lower productivity, particularly in cold-sensitive cultivars. CS can occur at different developmental stages and is generally classified into two types: delayed-type and sterility-type [6,7]. Sterilized-type chilling occurs during the booting and flowering stages, where it disrupts microspore development, resulting in a lack of viable pollen during anthesis and a subsequent decline in yield [3,8,9]. Delayed-type chilling affects the entire rice growth cycle, causing delays in maturity.

In the Yangtze River region, cold-induced damage has been linked to reduced rice yields. Longer-duration rice varieties, which are commonly grown in colder regions, have seen their suitable cultivation zones shift northward due to global warming, expanding the safe growing areas for these varieties [10]. However, this shift, along with the renewal of rice varieties, suggests that rice cultivation will increasingly face frequent and severe extreme weather events [11,12]. Delayed-type chilling poses a major challenge to increasing rice yields by restricting both the area available for cultivation and the length of the growing season. Severe CS during the growing season can reduce yields by up to 20% [13]. Key factors contributing to yield losses from delayed-type chilling include the timing and duration of CS, growth conditions, and agronomic management [14]. The tillering stage is particularly critical, as chilling during this period directly affects the development of rice stems, leaves, roots, tiller formation, flowering, maturity, and final yield [15].

Plant nanobiotechnology is an emerging field in agricultural research [16,17] and has shown potential to enhance plant performance under stress conditions (biotic and abiotic) [18]. A key question in agricultural nanobiotechnology is why nanomaterials should be integrated into crop production systems. Conventional agriculture often relies on increased agrochemical inputs to improve productivity; however, higher application rates do not necessarily translate into proportional yield gains and may even reduce crop performance due to nutrient imbalances, environmental contamination, and phytotoxic effects [19]. Furthermore, climate change is amplifying the frequency of abiotic and biotic stress, adding further complexity to agricultural challenges. For example, a field study demonstrated that iron oxide nanoparticles (10 mg/kg soil) reduced greenhouse gas emissions in paddy soils and significantly boosted rice yields by 23.6–54.9% [20]. However, field studies remain limited, and many investigations have primarily focused on pot experiments. Silica nanoparticles (400 mg/L, 2000 mg/L, and 4000 mg/L) stimulated antioxidant enzyme production and enhanced chlorophyll synthesis, in maize crop as compared to cold stress [21]. Additionally, carbon dots (5 mg/kg soil) increased protein, fatty acid, and amino acid concentrations in soybean grains under abiotic stress by 3.4%, 6.9%, and 17.3%, respectively, by enhancing nitrogen bioavailability [22].

Iron oxide Fe_2_O_3_ nanoparticles enhance plant physiological performance by supplying bioavailable iron required for chlorophyll biosynthesis, photosynthetic electron transport, and antioxidant defense [23]. Zinc oxide (ZnO) nanoparticles provide bioavailable zinc, promoting enzyme activity, protein synthesis, and antioxidant defense. In contrast, cerium oxide (CeO_2_) nanoparticles exhibit strong redox activity, efficiently scavenging reactive oxygen species (ROS) and mitigating oxidative damage in plants [24]. Titanium dioxide (TiO_2_) NPs have been shown to enhance photosynthetic efficiency by improving light absorption and electron transport rates. Under stress conditions, TiO_2_ NPs can scavenge ROS through their redox-active surface properties and have been reported to increase the activity of antioxidant enzymes in various crop species [25]. Despite these beneficial effects, the application of TiO_2_ NPs for cold stress mitigation in rice remains largely unexplored, warranting its inclusion in our comparative analysis. Together, these nanoparticles (NPs) improve nutrient use efficiency, strengthen antioxidant systems, and enhance plant tolerance to abiotic stresses such as cold stress. Compared to traditional agrochemicals, NPs offer advantages such as controlled nutrient release, improved plant growth promotion, greater nutrient use efficiency, and reduced environmental impact. Therefore, NPs hold promise for more effectively increasing crop productivity under environmental stress conditions than conventional agrochemicals.

In this study, CS treatments were conducted under controlled environmental conditions using a growth chamber programmed to maintain a precise temperature regime of 14 °C during the light period and 10 °C during the dark period for 5 days. Prior to stress imposition and during the recovery period, plants were maintained under natural field conditions at the experimental station of Huazhong Agricultural University, Wuhan, China (114.37° E, 30.48° N). This experimental design allowed us to combine the precision of controlled stress application with the ecological relevance of field-grown conditions during the recovery and yield assessment phases.

This study explores the potential of NPs to enhance rice resilience to CS during early seedling stages, focusing on growth and yield outcomes in the Middle Reaches of the Yangtze River, China. The findings of this research could provide valuable insights for the rice-growing community in regions prone to frequent chilling events, helping them implement preventive strategies to sustain stable rice production.

## 2. Results

### 2.1. Meteorological Factors

**Figure 1:**
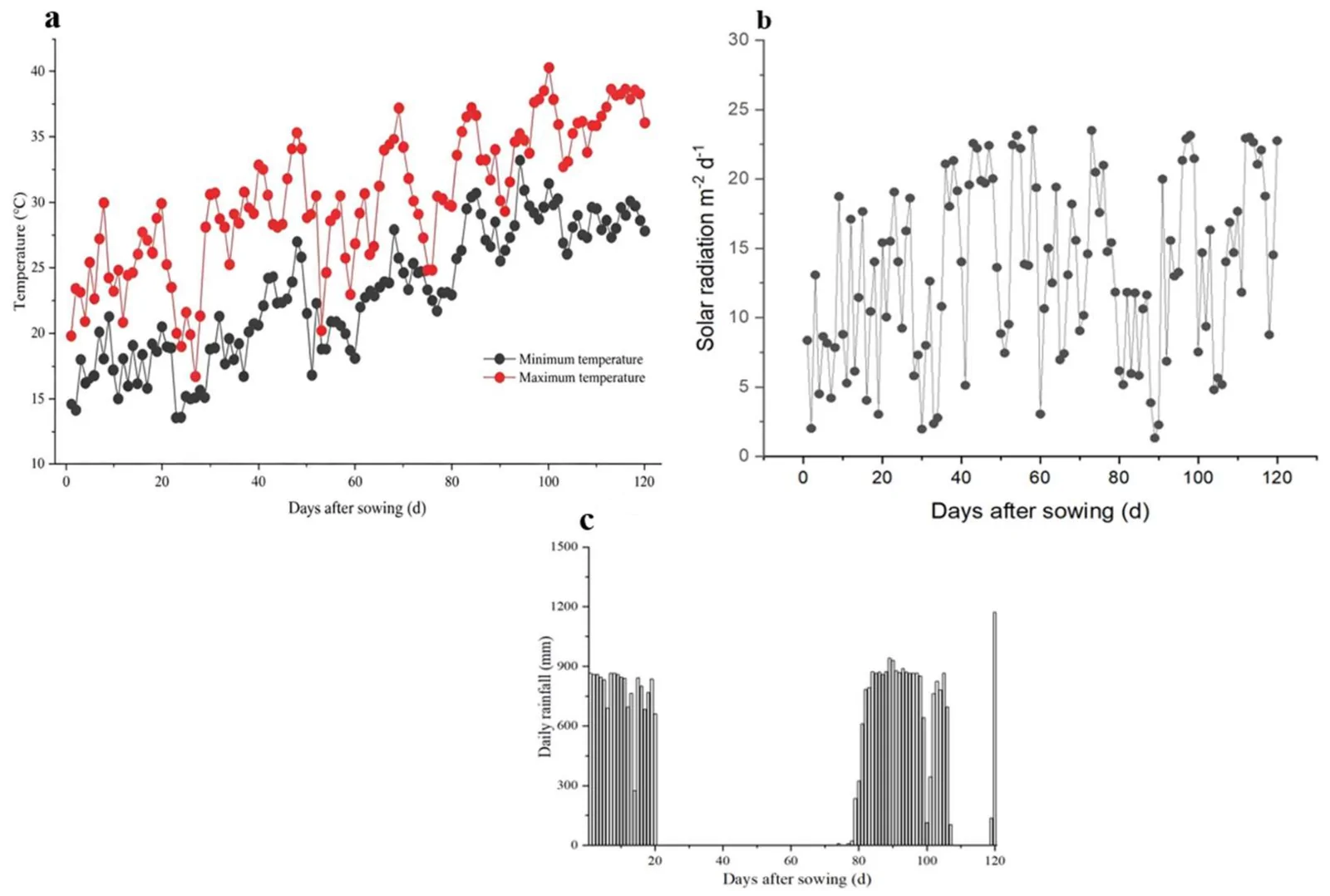
Daily average, minimum, and maximum temperatures (**a**), rainfall intensity and monthly cumulative rainfall (**b**), seasonal energy trends, and solar radiation (**c**) from 8 April to 8 August 2024.

From 8 April to 8 August 2024, temperature data show a peak maximum of 38.66 °C on 22 July, when the average temperature also reached 34.16 °C, while the lowest minimum (13.29 °C) occurred on 9 April, and the lowest maximum (16.7 °C) occurred on 4 May (Figure 1a). Rainfall patterns featured a dry spell from May to June, with events ranging from light (<100 mm) to very heavy (>1200 mm) (Figure 1b). Seasonal trends indicate a progressive decline in energy, with maxima falling from 23.5 MJ (12 June) to 2.35 MJ (3 May of the following year) (Figure 1c).

### 2.2. Effect of Nanoparticles on Plant Growth Parameters

Nanoparticles (NPs) had shown significant potential to improve crop growth under various stress conditions. NPs hold potential as a tool to address challenges in modern agriculture, especially in light of climate change, increasing population, and the need for sustainable practices. NPs application significantly (*p* < 0.05) influenced the plant height compared to the cold stress (CS) (Table 1). The application of NPs significantly ameliorated the negative effects of CS, resulting in taller plants compared to those subjected to CS alone. The decrease in the plant height due to CS was 34.8%. The plant height increased by 25.6% due to Fe_2_O_3_ (50 mg L^−1^) application, while foliar application of ZnO nanoparticles improved plant height as compared to CS, CeO_2_ and TiO_2_ application by 16.0% and 7.7%, respectively. Among the different cultivars, LLY-7108 performs best at both intervals as compared to other cultivars. The maximum plant height was recorded for LLY-7108 (35.3 cm), while the lowest average plant height recorded was 29.3 cm for ZJZ-17 across different growth stages. This decrease in plant height due to CS for ZJZ-17 was 16.99% as compared to the tolerant cultivar, LLY-7108. The data in Table 1 regarding fresh weight revealed the potential of nanoparticles to significantly alter the fresh weight of rice seedlings at different growth stages under CS. The decrease in the fresh weight of rice seedlings across three different growth stages due to CS was 57.2% as compared to CK (normal temperature). The foliar application of Fe_2_O_3_ increased the fresh weight of rice seedlings by 43.5%. Foliar application of ZnO, CeO_2_ and TiO_2_ reduced the adverse effect of CS on fresh weight by 33.7%, 24.4% and 14.5%, respectively. The tested cultivars have different degrees of tolerance to CS; LLY-7108 was found the most tolerant cultivar as compared to other varieties. The highest average fresh weight for LLY-7108 across three growth stages was 16.5% and 15.6% more compared to ZJZ-17 and LLY-32, respectively. However, the response of XZX-6 was found to be statistically similar to LLY-7108. The higher fresh weight was recorded at the third stage of cold stress (28 days) due to the period of more growth. The dry weight (g/plant) of rice seedlings was significantly (*p* < 0.05) influenced by CS and NPs application; similarly, the varieties respond differently to CS and NPs application across different growth stages (Table 1). The decrease in the dry weight of rice seedlings across three different growth stages due to CS is 50.0% as compared to CK (normal temperature). The foliar application of Fe_2_O_3_ increased the dry weight of rice seedlings by 40.6% across three stages as compared to CS. Foliar application of ZnO, CeO_2_ and TiO_2_ significantly recovered the reduction in dry weight due to CS. The increase in seedling dry weight due to foliar application of ZnO, CeO_2_ and TiO_2_ was 34.5%, 26.9% and 13.6%, respectively. Among the tested cultivars, LLY-7108 and XZX-6 perform best in terms of dry weight as compared to other varieties. The highest average dry weight for LLY-7108 across three growth stages was 3.0 g after a recovery period followed by a conventional variety, XZX-6. Similarly, the lowest average dry weight of 1.57 g was recorded after stress, while after 7 d of recovery the dry weight for ZJZ-17 was 2.5 g across different growth stages. The higher dry weight was recorded at the third stage of CS (exposure of 28 days seedling to CS) due to the period of more growth.

### 2.3. Influence of Nanoparticles on Photosynthetic Pigments

Nanoparticles (NPs) application significantly (*p* < 0.05) increased the chlorophyll a and chlorophyll b compared to the CS (Table 1). The application of NPs, especially Fe_2_O_3_ and ZnO, had a significant positive effect on chlorophyll a and b under cold stress, particularly improving pigment retention and recovery after stress. The decrease in chlorophyll a due to CS is 47.9% as compared to CK after a 7d recovery period. The chlorophyll a content increased by 41.6% due to Fe_2_O_3_ (50 mg L^−1^) application as compared to CS. Foliar application of other NPs, i.e., ZnO, TiO_2_ and CeO_2_, reduced the adverse effect of CS on chlorophyll a content. The increases in chlorophyll a due to ZnO, TiO_2_ and CeO_2_ foliar application were 36.6%, 19.3% and 29.5%, respectively, as compared to CS without NPs. Among the cultivars, a hybrid LLY-7108 performed best in terms of chlorophyll a as compared to other varieties. The maximum chlorophyll a content (174.6 Ug/g FW) was recorded in LLY-7108 after the recovery period. In terms of chlorophyll a, LLY-7108 was followed by a conventional variety, XZX-6 (165.9 Ug/g FW), while the lowest average chlorophyll a content (147.5 Ug/g FW) after recovery was recorded in ZJZ-17 across different growth stages. The exposure of rice seedlings to CS at different growth stages differentially influenced the photosynthetic pigments. The higher (165 Ug/g FW) photosynthetic a pigment was recorded at the third stage of CS (exposure of 28 days seedling to CS) exposure. The interactive effect showed that chlorophyll a content declined sharply under CS in all cultivars. Nanoparticle application restored chlorophyll a level, with Fe_2_O_3_ and CeO_2_ showing the strongest protective effects. The chlorophyll b content of rice seedlings was significantly (*p* < 0.05) influenced by CS and NPs application; similarly, the varieties respond differently to CS and NPs application in terms of chlorophyll b content (Table 1). The decrease in the chlorophyll b content of rice seedlings across three different growth stages due to CS is 48.0% after a 7d recovery period as compared to CK. The foliar application of Fe_2_O_3_ increased the chlorophyll b content of rice seedlings by 42.2% as compared to CS. Foliar application of ZnO, CeO_2_ and TiO_2_ significantly recovered the reduction in chlorophyll b content due to cold stress. The foliar application of ZnO, CeO_2_ and TiO_2_ increased the chlorophyll b content of rice seedlings by 34.6%, 24.3% and 14.3% as compared to CS. Among the tested cultivars, LLY-7108 performed best, with a maximum chlorophyll b content of 156.9 Ug/g FW, followed by a conventional variety, XZX-6. Similarly, the lowest average chlorophyll b content was recorded for ZJZ-17, which was 127.4 Ug/g FW across different growth stages. The exposure of rice seedlings to CS at different growth stages differentially influenced the photosynthetic pigments. The chlorophyll b content was recorded to be statistically similar for the second stage (exposure of 21 days seedling to CS) and third stage (exposure of 28 days seedling to CS). The higher (149.6 Ug/g FW) photosynthetic b pigment was recorded at the third stage of CS (exposure of 28 days seedling to CS). Nanoparticle treatments alleviated the decline in chlorophyll b due to CS, particularly Fe_2_O_3_, which resulted in the highest chlorophyll b accumulation.

### 2.4. Influence of Nanoparticles on ROS, MDA and Proline Content Under Cold Stress at Different Growth Stages

**Figure 2:**
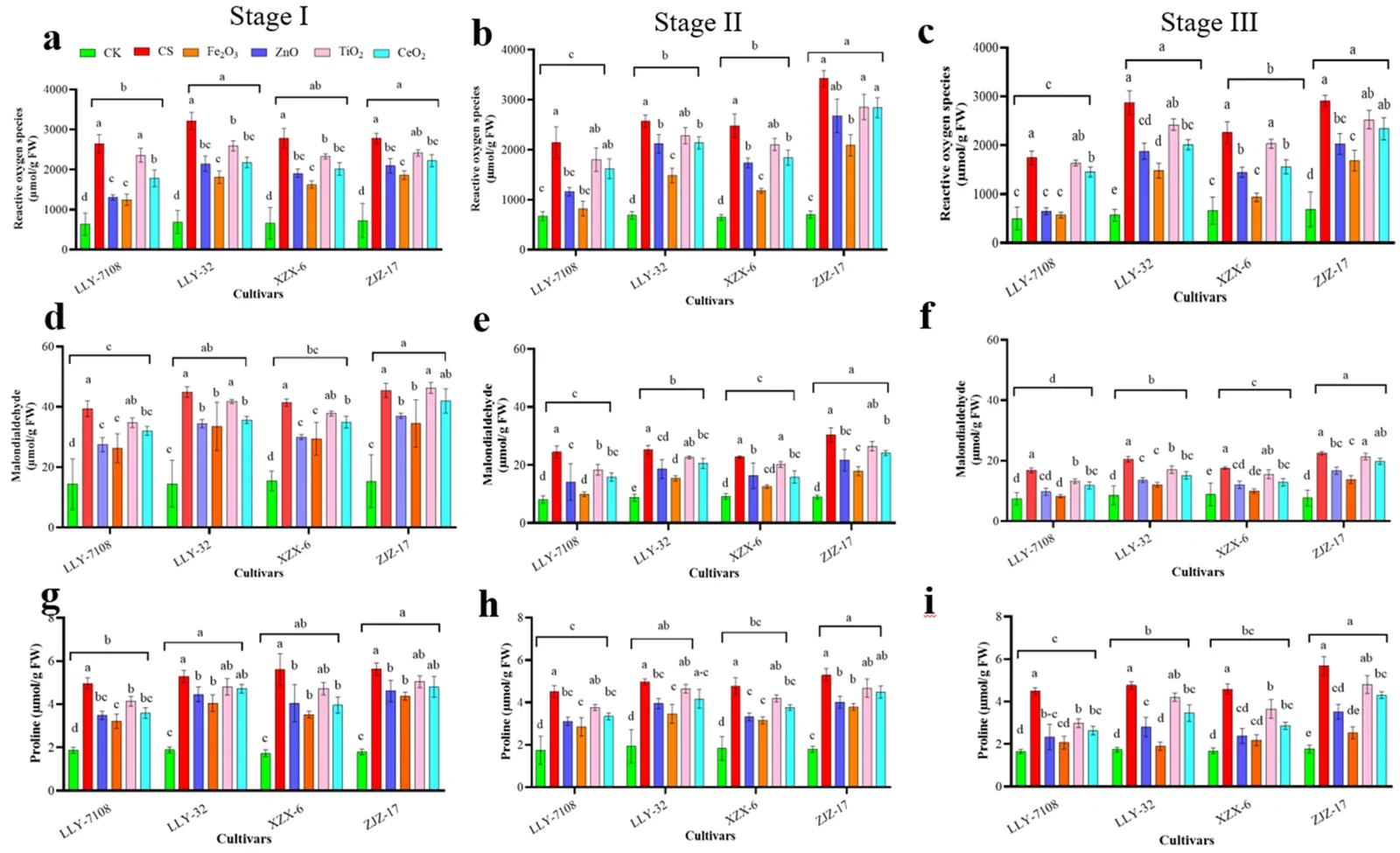
Influence of nanoparticles on reactive oxygen species (ROS) (**a**–**c**), MDA (**d**–**f**) and proline content (**g**–**i**). Data are presented as mean ± standard error (SE). Different lowercase letters above bars indicate significant differences among treatments within the same cultivar at *p* < 0.05. Brackets denote overall significant differences among cultivars.

Nanoparticles (NPs) have shown significant potential to reduce the oxidative stress and membrane damage in rice caused by cold stress (CS). ROS levels increased by 3.1-fold in LLY-7108, 3.7-fold in LLY-32, 3.21-fold in XZX-6, and 2.8-fold in ZJZ-17 (Figure 2a), indicating severe oxidative stress under low temperature. Foliar nanoparticle treatments reduced ROS accumulation to varying extents. Applications of Fe_2_O_3_ and ZnO were the most effective, reducing ROS by 31.7–41.6% and 25.7–50.6%, respectively, across cultivars. CS markedly increased ROS accumulation in all rice cultivars compared with CK. ROS levels increased by 2.2-fold in LLY-7108, 2.7-fold in LLY-32, 2.9-fold in XZX-6, and 3.9-fold in ZJZ-17 (Figure 2b), indicating severe oxidative stress at the second growth stage. Application of nanoparticles significantly reduced ROS levels relative to cold stress alone. In LLY-7108, ROS decreased by 45.8% (ZnO), 61.6% (Fe_2_O_3_), 16.6% (TiO_2_), and 17.0% (CeO_2_). Similar trends were observed in LLY-32, where ROS declined by 17.6–42.2%, with Fe_2_O_3_ showing the strongest mitigation. In XZX-6, ROS was reduced by 30.0–52.4%, while in the highly sensitive genotype ZJZ-17, reductions ranged from 16.6% (TiO_2_) to 39.0% (Fe_2_O_3_). Overall, Fe_2_O_3_ nanoparticles were most effective in suppressing cold-induced ROS accumulation. The exposure of rice seedlings to CS after 28 days of growth increased ROS levels by 2.5-fold in LLY-7108 and 3.2-fold in ZJZ-17, indicating severe oxidative stress induced by low temperature (Figure 1c). Application of nanoparticles markedly reduced ROS accumulation relative to CS alone. Under ZnO treatment, ROS decreased by 62.8% (LLY-7108), 34.6% (LLY-32), 36.0% (XZX-6), and 30.4% (ZJZ-17). Foliar application of Fe_2_O_3_ showed the strongest ROS-scavenging effect, reducing ROS by 67.4%, 48.5%, 58.7%, and 42.1%, respectively (Figure 2c).

MDA increased by 1.74–1.97-fold across cultivars under CS, confirming substantial membrane damage. Relative to CS, nanoparticle application significantly reduced MDA accumulation. Fe_2_O_3_ and ZnO treatments caused the greatest reductions (20.0–28.9% and 19.4–27.5%, respectively), followed by CeO_2_ (11.8–22.9%). In contrast, TiO_2_ showed minimal or inconsistent mitigation and, in ZJZ-17, slightly increased MDA relative to CS, indicating limited protective capacity against membrane damage (Figure 2d). In the second growth stage, CS significantly enhanced lipid peroxidation, compared with CK, and MDA increased by 2.02 (LLY-7108), 1.86-fold (LLY-32), 1.47-fold (XZX-6), and 2.42-fold (ZJZ-17). Nanoparticle application markedly alleviated membrane damage. Relative to CS, MDA content decreased by 42.4–59.6% in LLY-7108, 26.2–38.9% in LLY-32, 28.2–44.5% in XZX-6, and 13.0–41.1% in ZJZ-17 (Figure 2e). The exposure of rice seedlings to CS after 28 days of growth increased membrane damage relative to CK, and increased MDA by 1.27-fold in LLY-7108, 1.38-fold in LLY-32, 1.0-fold in XZX-6, and 1.96-fold in ZJZ-17. Nanoparticle application significantly mitigated membrane damage under CS. ZnO reduced MDA by 42.3%, 33.7%, 31.8%, and 25.8% in LLY-7108, LLY-32, XZX-6, and ZJZ-17, respectively. Fe_2_O_3_ resulted in stronger reductions (51.2–43.2%), while TiO_2_ and CeO_2_ also decreased MDA accumulation (Figure 2f).

Compared with CK, CS induced a strong accumulation of proline, with increases of 1.63–2.3-fold, reflecting enhanced osmotic adjustment under cold stress conditions (Figure 2g). Treatments of different nanoparticles generally reduced proline levels, suggesting alleviation of stress intensity. ZnO and Fe_2_O_3_ resulted in 14.3–37.5% and 21.4–37.5% reductions, respectively. In the second growth stage of rice seedlings, NPs treatments significantly reduced proline levels relative to CS, suggesting stress alleviation. Proline content declined by 11.3–31.1% in LLY-7108, 8.0–30.0% in LLY-32, 12.5–33.3% in XZX-6, and 11.3–28.3% in ZJZ-17. The largest reductions were generally observed with Fe_2_O_3_ and ZnO, indicating reduced cellular stress under nanoparticle supplementation (Figure 2h). CS induced a substantial accumulation of proline compared with CK, with increases of 1.65-fold (LLY-7108), 1.82-fold (LLY-32), 1.70-fold (XZX-6), and 2.17-fold (ZJZ-17), reflecting osmotic adjustment under stress conditions. Compared with CS, nanoparticle treatments significantly lowered proline content. ZnO reduced proline by 41.7–48.9%, while Fe_2_O_3_ showed the greatest decrease (52.2–60.4%). TiO_2_ and CeO_2_ treatments also reduced proline accumulation by 12.5–33.3% and 24.6–42.2%, respectively, indicating alleviation of cold-induced osmotic stress. Additionally, the results of the regression analysis (Figure S1) revealed that the MDA and ROS content was negatively correlated with shoot growth including fresh weight, dry weight, and length, suggesting the more membrane damage and ROS content, the less shoot growth.

### 2.5. Influence of Nanoparticles on Antioxidant Enzymatic Activity Under Cold Stress

**Figure 3:**
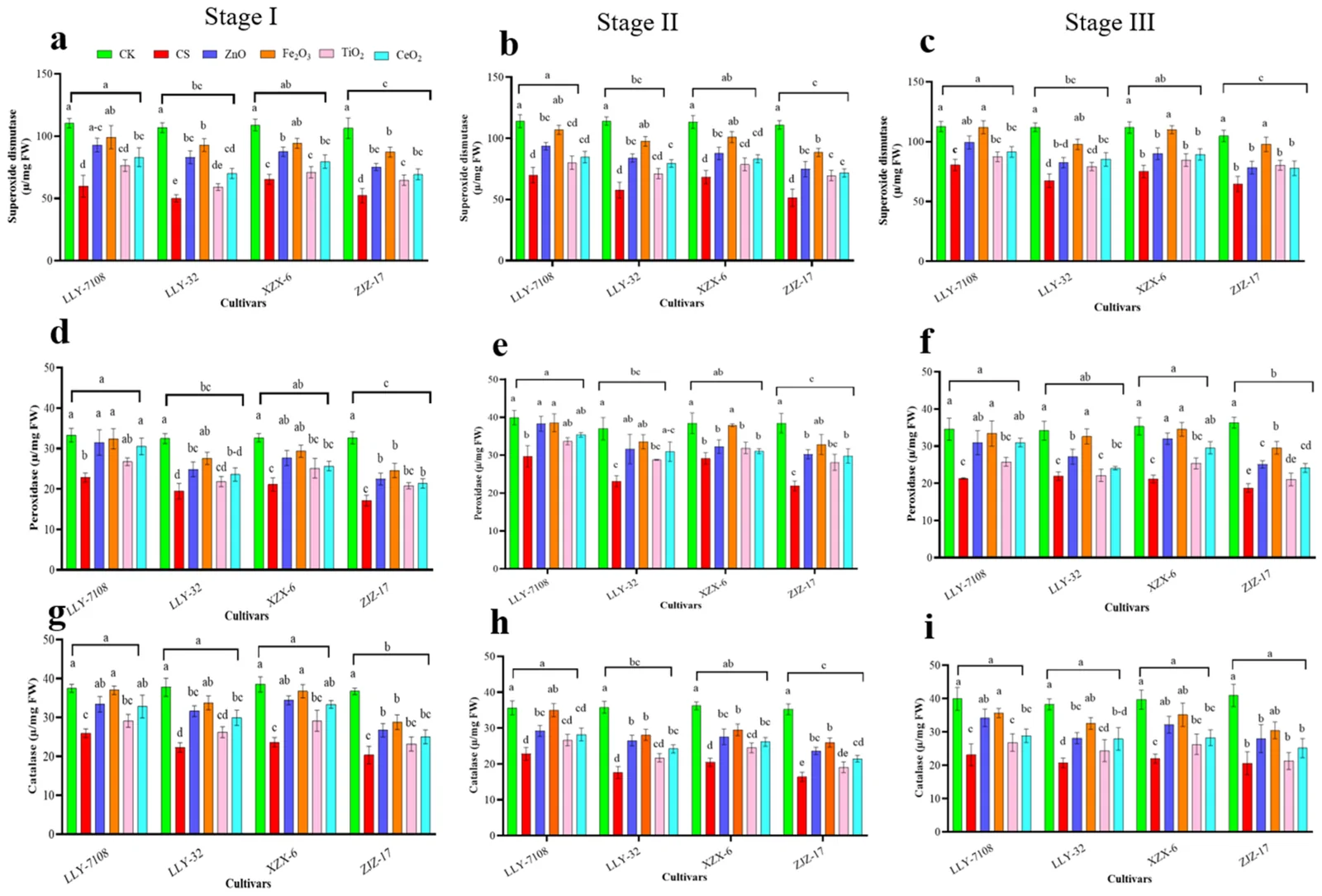
Influence of nanoparticles on SOD (**a**–**c**), POD (**d**–**f**) and CAT (**g**–**i**) content. Data are presented as mean ± standard error (SE). Different lowercase letters above bars indicate significant differences among treatments within the same cultivar at *p* < 0.05. Brackets denote overall significant differences among cultivars.

Cold stress (CS) significantly suppressed SOD activity relative to CK, with reductions of 45.8–53.0% across cultivars, demonstrating impaired primary antioxidant defense (Figure 3a). Compared to CS, nanoparticle application markedly restored SOD activity. Fe_2_O_3_ showed the strongest enhancement, increasing SOD by 44.3–66.6%, followed by ZnO (34.2–58.7%) and CeO_2_ (32.8–50.6%) (Figure 3a). Nanoparticle application substantially restored SOD activity in the second growth stage under CS. Relative to CS, SOD activity increased by 33.8–53.0% in LLY-7108, 23.8–69.6% in LLY-32, 29.5–48.1% in XZX-6, and 35.9–72.0% in ZJZ-17. Foliar Fe_2_O_3_ treatment consistently induced the highest SOD enhancement, highlighting its strong role in strengthening antioxidant defense (Figure 2b). CS markedly suppressed SOD activity relative to CK in the third growth stage, with reductions of 28.7% (LLY-7108), 40.0% (LLY-32), 32.9% (XZX-6), and 38.5% (ZJZ-17). Nanoparticle application significantly enhanced SOD activity compared with CS. ZnO increased SOD by 19.9–23.6%, while Fe_2_O_3_ produced the highest stimulation (39.3–51.5%). TiO_2_ and CeO_2_ treatments also improved SOD activity, with increases ranging from 8.6 to 24.3% and 13.7 to 26.6%, respectively (Figure 3c).

CS for 5 days significantly reduced peroxidase (POD) activity in all rice cultivars at the three developmental stages; however, nanoparticle application markedly alleviated this reduction in a stage- and cultivar-dependent manner (Figure 3d,e). Overall, Fe_2_O_3_ and ZnO were more effective in restoring POD activity than TiO_2_ and CeO_2_, with stronger responses observed at later growth stages. At the first growth stage (14-day-old seedlings; Figure 3d), CS caused a pronounced decline in POD activity across all cultivars compared with the CK. The reduction was most evident in ZJZ-17, indicating higher sensitivity at this early stage. Application of nanoparticles significantly enhanced POD activity relative to CS in all cultivars. Among treatments, Fe_2_O_3_ resulted in the greatest recovery of POD activity, often restoring values close to CK, whereas TiO_2_ and CeO_2_ produced moderate but significant increases. At the second growth stage (21-day-old seedlings; Figure 3e), CS again significantly suppressed POD activity compared with CK, though the magnitude of reduction was slightly lower than that observed at the first stage. Nanoparticle application effectively counteracted this decline, with Fe_2_O_3_ consistently inducing the highest POD activity, followed by ZnO, TiO_2_ and CeO_2_ treatments. Notably, cultivars LLY-7108 and XZX-6 showed a stronger recovery of POD activity than LLY-32 and ZJZ-17, suggesting cultivar-specific tolerance mechanisms at this developmental stage. At the third growth stage (28-day-old seedlings; Figure 3f), POD activity under CK was generally higher than at earlier stages, while CS still caused a significant reduction across all cultivars. However, nanoparticle application at this stage produced the most pronounced alleviation of cold-induced inhibition. Fe_2_O_3_ and ZnO treatments restored POD activity to levels comparable to or approaching CK, particularly in LLY-7108 and XZX-6. Collectively, these results demonstrate that cold stress for 5 days significantly impairs POD activity at all growth stages, with the greatest sensitivity observed at the early seedling stage. Nanoparticle application, especially Fe_2_O_3_ and ZnO, effectively mitigates cold-induced oxidative stress by enhancing POD activity, and this protective effect becomes more pronounced as seedlings advance in growth.

The catalase activity was significantly (*p* < 0.05) increased by foliar application of nanoparticles, while CS reduces the catalase activity; similarly, the varieties respond differently. The catalase (CAT) activity decreases with CS; however, NPs application under CS increases the CAT activity. CS to rice seedlings diminished the CAT activity by 43.5% as compared to CK across different developmental stages and cultivars. The foliar application of Fe_2_O_3_ boosted the CAT activity of rice seedlings by 34.3% as compared to CS seedlings (Figure 3g–i). Foliar application of ZnO, CeO_2_ and TiO_2_ significantly (*p* < 0.05) increased the CAT activity in rice seedlings. Among the tested cultivars, the hybrid LLY-7108 showed increased CAT activity under CS and performed best against CS as compared to other varieties. The maximum CAT activity for LLY-7108 across three growth stages of 32.1 U/mg FW was observed in LLY-7108, as compared to the lowest CAT activity (26.0 U/mg FW) observed in ZJZ-17. The growth stages of rice differently respond in terms of CAT activity. The CAT activity in the second stage, i.e., exposure of 21-day seedlings to CS, was found to be the minimum (26.6 U/mg FW), while the CAT activity was found to be the maximum (29.6 U/mg FW) in the first crop growth stage, i.e., exposure of 28-day seedlings to CS (Figure 3g–i). Additionally, regression analysis (Figure S2) showed that SOD, POD, and CAT activities were positively correlated with shoot growth parameters, including fresh weight, dry weight, and shoot length, indicating that enhanced antioxidant enzyme activity is associated with improved shoot growth.

### 2.6. Yield and Yield Components of Rice Influenced by Cold Stress and Nanoparticles Application in Seedling Stages

**Figure 4:**
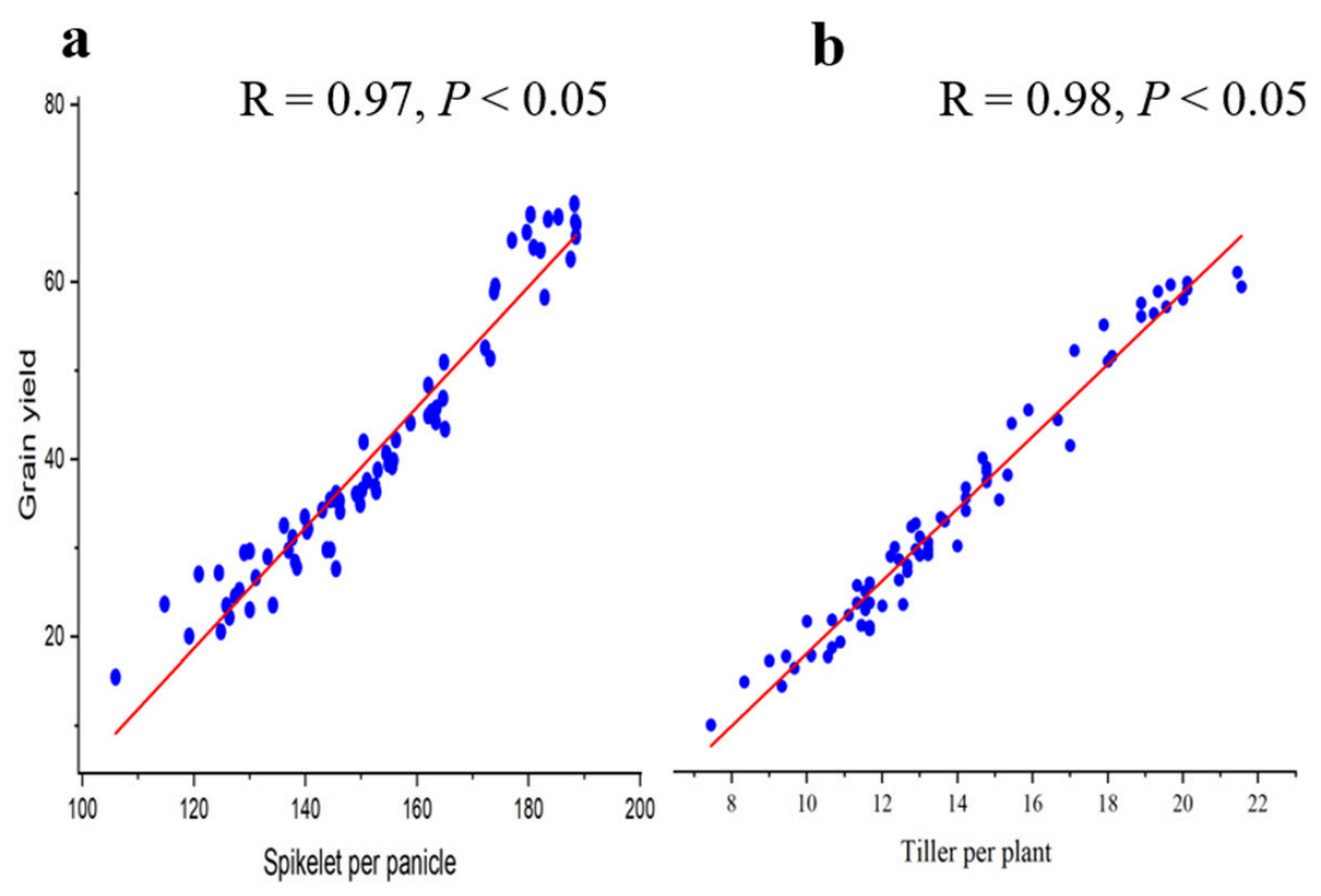
This figure shows two scatter plots with regression lines demonstrating relationships between grain yield and two agronomic traits. (**a**) Grain yield vs. Spikelets per panicle; the data points (blue dots) show a strong positive linear relationship: as spikelet number per panicle increases, grain yield also increases. The correlation coefficient is R = 0.97, which indicates a very strong correlation. (**b**) Grain yield vs. Tiller per plant; a strong positive linear relationship: plants with more tillers produce higher grain yield. The correlation coefficient is even stronger (R = 0.98), suggesting an almost perfect linear relationship.

Cold stress (CS) at the seedling stage significantly (*p* < 0.05) influences rice yield and yield component (Table 2). CS significantly diminishes the yield and yield traits while NPs recover the reduction in yield and yield trait. CS and NPs application significantly influence tiller per plant; however, varieties exhibit differentially under cold stress (Table 2). NPs foliar application shows a positive effect on tillers per plant under CS. The maximum average tiller per plant (19.6) was observed under CK; where CS reduces tillers per plant (10.5), the reduction due to CS is 46.4%. The NPs application shows a promising effect on tillers per plant; the most effective nanoparticle was Fe_2_O_3_, which reduced the adverse effect of CS and increased the number of tillers per plant (15.3), followed by ZnO application (13.6). Among the tested nanoparticles, the lowest tiller number per plant (11.6) was observed with the TiO_2_ application. Among the cultivated varieties, LLY-7108 (hybrid) showed tolerant behavior and had more tillers per plant (14.5) as compared to any other variety. The minimum number of tillers per plant (13.1) was observed with ZJZ-17. In terms of different growth stages, CS at the third stage, i.e., CS after 28 days of emergence, has minimal effect on tiller numbers per plant, while the maximum reduction in tiller numbers was recorded in the first growth stage, i.e., cold stress after 14 days of emergence. CS reduced the tiller number at all growth stages. Nanoparticle application significantly increased tillering compared with CS alone, with the most notable enhancement at stage II. CS treatment on different growth stages showed significant effects on spikelet per panicle while thousand grain weight was found to be non-significant (*p* < 0.05) (Table 2). NPs foliar application shows promising effect on spikelet per panicle under CS treatments. The maximum spikelets per panicle was recorded under normal temperature (184.5), where CS reduced spikelets per panicle (127.2). The most effective nanoparticle was Fe_2_O_3_, which reduced the adverse effect of CS and increased the number of spikelets per panicle (163.7), followed by ZnO application (152.5), which was statistically similar to CeO_2_ application. Among the tested NPs, the lowest spikelets per panicle (139.6) was observed with the TiO_2_ application. Among different cultivars, LLY-7108 (hybrid) show tolerant behavior and had the highest spikelets per panicle (160.0), followed by the conventional variety XZX-6. The minimum number of spikelets per panicle (144.0) was observed with ZJZ-17. The CS induces minimum damage to rice crop in terms of spikelet per panicle, when the crop is exposed to CS at the third growth stage, i.e., 28 days after emergence. Maximum spikelets per panicle (160.0) was recorded at the third growth stage, followed by the second growth stage (152.7). CS treatment and NPs application in the seedling stage have promising effects on rice yield (Table 2). CS treatment significantly reduces the yield of rice per plant; the maximum yield (65.7 g/plant) was recorded with normal temperature, while CS reduces the productivity of rice to 25.3 g/plant. NPs foliar application increases rice yield under CS. The maximum yield per plant (48.2 g/plant) was recorded with Fe_2_O_3_ foliar application; this increase in productivity is 47.5% as compared to the yield reduced by CS. The second most effective nanoparticles are ZnO and the yield increase due to zinc oxide foliar application is 35.6% as compared to CS. In terms of NPs application, the lowest yield (30.1 g/plant) was measured in the plot sprayed with TiO_2_. Among the cultivated varieties, LLY-7108 (hybrid) showed tolerant behavior; the yield production per plant was higher (44.1 g/plant) as compared to any other variety. The hybrid LLY-32 and conventional variety ZJZ-17 showed statistically similar responses across different treatments. The minimum yield per plant (36.3 g/plant) was observed with ZJZ-17. The CS induces minimum damage to the rice crop in terms of yield production, when the crop is exposed to cold stress at the third growth stage, i.e., 28 days after emergence. The maximum yield per plant (46.1 g/plant) was recorded at the third growth stage, followed by the second growth stage (40.4 g/plant), while the minimum yield per plant (35.2 g/plant) was recorded when the crop was exposed to CS at early stages. Grain yield was severely reduced under CS. Nanoparticle application significantly improved yield, particularly at the third stage, with Fe_2_O_3_ treatment producing the highest grain yield. The liner regression analysis indicates a strong correlation among spikelet per panicle with grain yield and tiller per plant with grain yield; plants with more tillers and higher spikelet per panicle produce higher grain yield (Figure 4).

### 2.7. Cultivar-Specific Responses to Nanoparticle Treatments Under Cold Stress

The four rice cultivars exhibited differential responses to NP treatments under cold stress, with distinct patterns of recovery across morphological, physiological, and yield parameters (Table 1 and Table S1).

LLY-7108 (cold-tolerant hybrid) demonstrated the highest overall tolerance and greatest responsiveness to NP application. Under CS alone, this cultivar maintained relatively higher chlorophyll content (1.8-fold higher than ZJZ-17), lower ROS accumulation (2.2-fold increase vs. 3.9-fold in ZJZ-17), and superior membrane stability (MDA: 1.27-fold increase vs. 2.42-fold in ZJZ-17). Following Fe_2_O_3_ treatment, LLY-7108 showed maximum recovery in plant height (28.4%), fresh weight (46.2%), and grain yield (95.6% increase over CS). The hybrid vigor of LLY-7108, combined with its robust antioxidant system, enabled greater NP uptake and utilization efficiency.

XZX-6 (cold-tolerant conventional) exhibited similar tolerance patterns to LLY-7108, though with slightly lower recovery magnitudes. Under CS, XZX-6 maintained moderate chlorophyll retention and antioxidant enzyme activities (SOD reduction: 46.2% vs. 53.0% in ZJZ-17). Fe_2_O_3_ treatment enhanced plant height by 26.1%, fresh weight by 44.8%, and grain yield by 88.3% compared to CS. XZX-6 showed comparable responsiveness to ZnO and CeO_2_ treatments, though overall recovery was marginally lower than LLY-7108.

LLY-32 (cold-sensitive hybrid) displayed moderate tolerance with limited NP responsiveness. Despite being a hybrid, LLY-32 showed greater stress susceptibility than LLY-7108, with higher ROS (3.4-fold increase) and MDA accumulation (2.18-fold increase) under CS. Fe_2_O_3_ treatment improved plant height (24.3%), fresh weight (41.7%), and grain yield (75.4% over CS), but these recoveries were significantly lower than those observed in tolerant cultivars. The moderate response suggests that genetic factors beyond hybrid status influence NP efficacy.

ZJZ-17 (cold-sensitive conventional) was the most susceptible cultivar, exhibiting the highest oxidative damage (ROS: 3.9-fold; MDA: 2.42-fold) and greatest reductions in antioxidant enzyme activities (SOD: 53.0%; POD: 52.0%) under CS. NP treatments showed limited effectiveness in this cultivar, with Fe_2_O_3_ improving plant height by only 21.8%, fresh weight by 38.2%, and grain yield by 65.7% over CS. The poor response is attributed to compromised membrane integrity, reduced NP uptake, and inherently weak antioxidant capacity, limiting the cultivar’s ability to utilize NP-mediated protection.

### 2.8. Pearson Correlation and Structure Model Equation

**Figure 5:**
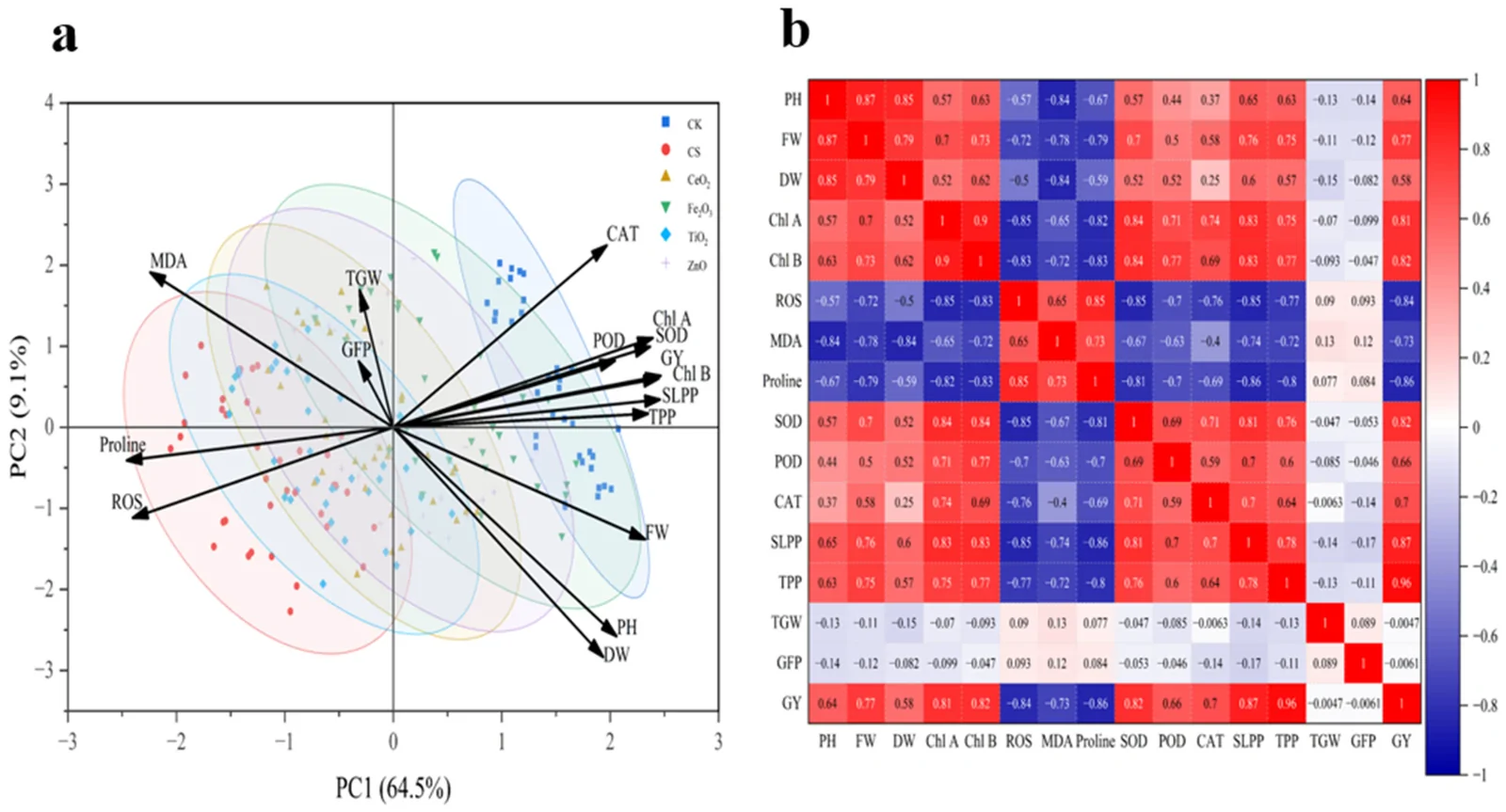
Multivariate relationships among growth, physiological, and biochemical traits of rice under cold stress and nanoparticle treatments. (**a**) Principal component analysis (PCA) biplot showing the distribution of treatments and the contribution of measured variables to PC1 (72.25%) and PC2 (9.46%). (**b**) Pearson correlation heatmap illustrating significant positive (blue) and negative (red) correlations among morphological, physiological, antioxidant, and yield-related parameters, highlighting the antagonistic relationship between oxidative damage markers and growth, antioxidant and yield traits.

**Figure 6:**
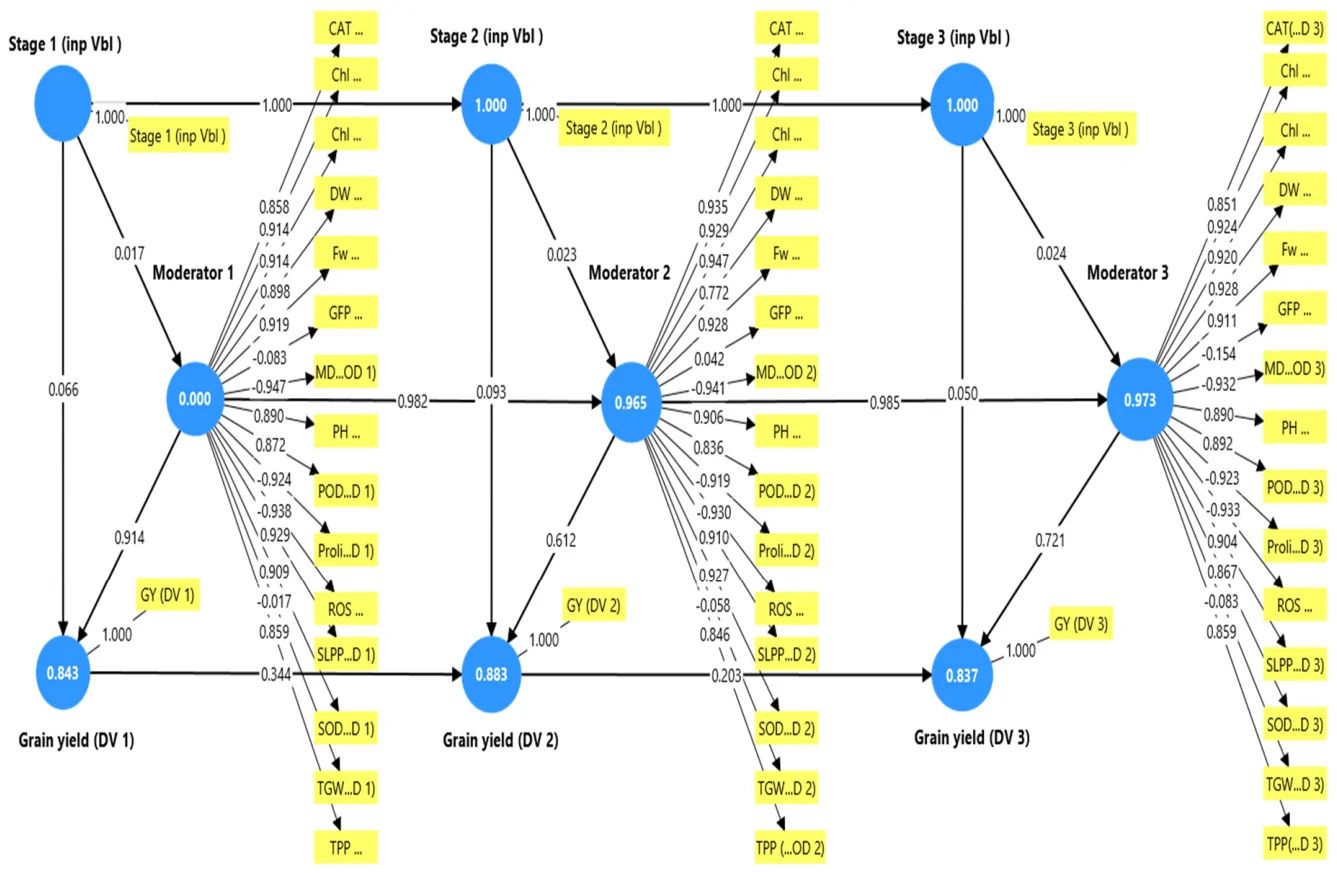
Structural equation model (SEM) illustrating the sequential relationships among rice developmental stages and their effects on physiological traits and grain yield under different treatments. Stage 1, Stage 2, and Stage 3 represent three consecutive growth stages of rice, while the nodes labeled Moderator 1, Moderator 2, and Moderator 3 are latent variables representing the integrated physiological status at each corresponding growth stage rather than statistical moderators. These latent variables summarize the combined effects of agronomic parameters and link the physiological responses across successive growth stages. Grain yield (GY) at each stage is modeled as the dependent variable. Numbers on the arrows represent standardized path coefficients, and values within the blue circles indicate the coefficient of determination (R^2^) for variables.

Principal component analysis (PCA) showed that PC1 and PC2 explained 72.25% and 9.46% of the total variance, respectively, clearly separating growth and antioxidant-related traits from oxidative stress indicators. Biomass traits, chlorophyll contents, antioxidant enzymes, and yield components were positively associated along PC1, whereas ROS, MDA, and proline loaded negatively, indicating their antagonistic effects (Figure 5a). Correlation analysis further confirmed strong positive relationships among growth, photosynthetic pigments, antioxidant activities, and grain yield, while oxidative stress markers exhibited significant negative correlations with these traits, highlighting the central role of redox homeostasis in yield performance under stress conditions (Figure 5b). Partial least squares structural equation modeling (PLS-SEM) revealed strong and consistent relationships between physiological traits and grain yield across all replications. The measurement model showed high indicator loadings (>0.85) for growth attributes, chlorophyll pigments, antioxidant enzymes, and yield components, confirming robust construct reliability. In contrast, oxidative stress markers (ROS and MDA) exhibited strong negative loadings, indicating their inhibitory effects on plant performance (Figure 6). The structural model demonstrated a pronounced sequential regulation, where Alpha strongly influenced Beta (path coefficient = 0.983), which in turn significantly affected Gamma (0.985). Gamma exerted a substantial positive effect on grain yield (0.721), highlighting the integrative role of antioxidant capacity, photosynthetic efficiency, and biomass accumulation in yield determination. The model explained a high proportion of variance in grain yield, with R^2^ values of 0.843, 0.883, and 0.837 for DV1, DV2, and DV3, respectively. The consistency of path coefficients and explanatory power across replications confirms the robustness of the proposed physiological framework underlying yield formation under stress conditions.

## 3. Discussion

Cold stress is a major environmental constraint during the early seedling stage of rice, severely limiting plant establishment and subsequent productivity. Exposure to low temperatures disrupts photosynthetic performance, impairs membrane integrity, disturbs cellular metabolism, and promotes excessive accumulation of reactive oxygen species (ROS), resulting in oxidative damage and growth inhibition. These physiological and biochemical disturbances collectively reduce seedling vigor and increase susceptibility to cold injury. Cold stress (CS) severely inhibited rice seedling growth, reducing plant height (34.8%), fresh weight (57.2%), and dry weight (50.0%) across all cultivars (Table 1), consistent with previous reports of impaired cell division and biomass accumulation under low temperatures [26,27]. Photosynthetic pigments declined markedly (chlorophyll a: 47.9%; chlorophyll b: 48.0%), indicating damage to chloroplast development and photosynthetic electron transport [28,29,30]. CS triggered excessive ROS accumulation (2.2- to 3.9-fold), with the cold-sensitive ZJZ-17 showing the highest increase and the tolerant LLY-7108 the lowest (Figure 2a–c), confirming genotype-dependent oxidative stress responses.

Membrane integrity was compromised, as evidenced by increased MDA content (1.47- to 2.42-fold; Figure 2d–f), with ZJZ-17 exhibiting the highest lipid peroxidation and LLY-7108 the lowest, supporting the superior membrane stability of cold-tolerant cultivars [31]. Proline accumulation increased (1.63- to 2.30-fold; Figure 2g–i), with sensitive genotypes showing higher accumulation, suggesting greater osmotic adjustment requirements in stress-susceptible cultivars [32,33]. Rice, a crop native to tropical and subtropical regions, exhibits high sensitivity to low-temperature stress. Consequently, sustained research efforts are essential to enhance our understanding of environmental stressors and to facilitate the development of improved cultivars for sustainable agricultural production. Plant nanotechnology is an emerging approach aimed at enhancing the functions of plant organelles, tissues, and entire organisms through the integration of nanomaterials [34,35]. This interdisciplinary field at the interface of nanotechnology and plant biology has the potential to augment tolerance to abiotic stress of plants by embedding nanoparticles (NPs) within photosynthetic tissues and organelles [34,36]. In different plants, the resistance to the stresses (biotic and abiotic) through application of different NPs has been reported [37,38]. However limited studies are available to address the influence of NPs on CS in rice. A single study from [26] addressed the effect of ZnO on CS at the seedling stage. However, a study from [39] addressed the influence of ZnO, Fe_2_O_3_, TiO_2_ and CeO_2_ nanoparticles under CS at the seedling stage. In the present work, we observed that the foliar treatment with ZnO, Fe_2_O_3_, TiO_2_ and CeO_2_ nanoparticles is effective in alleviating the CS in rice seedlings. Particularly, the Fe_2_O_3_ application largely enhanced resistance against CS in rice, restoring the chlorophyll content, decreasing the concentration of ROS, promoting the performance of antioxidant enzyme, and increasing yield and yield component. This study demonstrates that CS has a detrimental effect on the morphological parameters (e.g., plant height, fresh weight, and dry weight) of rice seedlings. Notably, the application of NPs under CS conditions resulted in significant increases in seedling height, fresh weight, and dry weight compared to treatments under CS. Consistent with previous findings of [36], foliar application of CSs alleviated cold-induced photosynthetic impairment and enhanced photoprotection, which collectively contributed to improved biomass accumulation under CS. This is due to the limited ability of rice seedlings to absorb nutrients, and reducing their assimilated production could be a key factor contributing to their slow recovery in terms of their fresh and dry weight [27,40]. The results of our experiment are consistent with [26], which found an increase in morphological characteristics in rice plants under CS with ZnO foliar application in hydroponic systems. The results of [39] are also in line with our results. In comparing different nanoparticles, Fe_2_O_3_ was found to be the most effective NP. This may be because of the increased photosynthesis rate, because iron is a component of cytochromes and ferredoxin, which are involved in the electron transport chain (ETC) of the photosynthetic light reactions [41,42]. Cytochromes (especially cytochrome b6f) are iron-containing heme proteins that mediate electron transfer between PSII and PSI, while Ferredoxin, a small iron–sulfur protein, accepts electrons from PSI and is involved in NADP^+^ reduction during the final stages of the light-dependent reactions [43,44,45]. In light of the above literature, it is a fact that foliar application of Fe_2_O_3_ can improve electron flow, leading to more efficient ATP and NADPH production for the Calvin cycle. The cultivar responds differently under CS; LLY-7108 (hybrid) had a better response to CS as compared to ZJZ-17 (conventional), which might be due to the poor CS tolerance of ZJZ-17. This result is consistent with [46], which related the poor performance of B144 to poor CS tolerance. CS triggers oxidative bursts, leading to the accumulation of signaling substances such as MDA and ROS [31,47,48,49], as well as compatible osmolytes like free proline [50]. However, these responses vary among different higher plants, showing dependence on factors such as dose, tissue type, developmental stage, and genotype. In rice, CS significantly boosts the oxidative damage [26,39] due to the reduction in the fluidity of cell membranes. Membranes, composed of lipids, become less flexible when exposed to low temperatures. This decrease in membrane integrity can result in leakage of cellular contents. The fluidity of membranes is essential for their functioning, including nutrient and water transport, protein insertion, and cellular communication. NPs, especially Fe_2_O_3_, increase the membrane fluidity and reduce membrane damage. This may be due to the reason that metal-based NPs can interact with membrane surfaces, helping to stabilize lipid bilayers and reducing permeability [51]. NPs may acquire a protein corona in planta, which mediates interactions with cell membranes either facilitating or impeding nanoparticle uptake and influencing membrane stability [32]. Under CS, certain rice cultivars exhibited increased concentrations of H_2_O_2_ and MDA, as well as compatible osmolytes, i.e., free proline under stress conditions [33,52]. In our study, the free proline, ROS and MDA content was found to be minimal in the CS tolerant cultivar, i.e., LLY-7108, while ZJZ-17 exhibited higher osmolyte content. In crop growth stages, cultivars exposed to the third stage of CS face minimal membrane damage, which might be due to the increased tolerance in the latter stage. CS and NPs application affect antioxidants, including SOD, CAT, and POD [53,54]. In our study the antioxidant enzyme activity reduced with CS; however, NPs application increased the antioxidant enzymatic activity. This reduction may be due to partial denaturation or misfolding of enzymes, altering their active sites due to CS. Many antioxidant enzymes require cofactors (e.g., Fe, Mn, Cu, Zn) or coenzymes (e.g., NADPH, ascorbate) and CS may impair cofactor biosynthesis, or cofactor stability, limiting enzyme activity [28,55]. Our results are in line with [26], which reported an increase in antioxidant enzymatic activity and gene expression due to ZnO application. This might be due to the fact that iron from Fe_2_O_3_ serves as an essential cofactor for Fe-dependent enzymes, including CAT and certain SOD isoforms; supplementing iron enhances the catalytic stability and turnover rate of these enzymes, particularly under stress conditions. CS has a detrimental impact on the metabolic and physiological functions of rice, leading to a decrease in yield by impeding the formation of chloroplasts and the biosynthesis of chlorophyll in leaves [29,30,56]. Most previous experimental research on direct seeded rice has evaluated the effects of chilling treatment temperature and duration at a particular growth stage on rice growth and yield [15,57,58,59]. However, no study analyzed the effects of CS and nanoparticle treatment at different phases on rice production. In this study we found that CS treatments at different phases have differential effects on yield and yield component, where NPs also significantly influence the yield and yield components. CS treatment reduces yield components, i.e., tillers per plant and spikelets per panicle and yield of rice cultivars [60], whereas NPs application significantly ameliorates the adverse effect of CS on rice yield and yield components. An earlier study by [61] found that exposing 24 japonica rice varieties to a continuous 10-day cold treatment at 15 °C during the tillering stage resulted in reduced grain yield. Delayed CS during the early and middle tillering stages primarily reduced rice grain yield by decreasing spikelets per panicle and tiller per plant [60]. Our study showed that even short-duration chilling (5 days) negatively impacted spike formation and tiller numbers. Foliar application of NPs significantly increased the yield and yield component of rice. This may be due to the chlorophyll accumulation and fast recovery after the stress period. Our results are in line with [62], which found that application of NPs increased the grain yield of cereals under stress conditions. Similarly, [63] showed the positive impacts of ZnO nanoparticle foliar spray on rice plants. In our study, we found that the early and middle tillering phases were particularly critical for spike development and tiller numbers. This may be due to the stunted growth and membrane damage due to cold stress. However, in contrast, a study by [6] indicated that the late tillering phase is very critical to low-temperature stress in terms of yield and yield component. In our study, CS treatment at the early seedling stage has no substantial effect on thousand grain weight and grain filling percentage. Variations in rice yield and its components also depend on genotype, growing-season climate, and farming practices [64,65].

## 4. Materials and Methods

### 4.1. Plant Materials and Growth Conditions

In this study, four early indica rice cultivars were used: Zhongjiazao-17 (ZJZ-17), Xiangzaoxian-6 (XZX-6), Lingliangyou-7108 (LLY-7108), and Lingliangyou-32 (LLY-32) (Table 3). ZJZ-17 is a cold-sensitive cultivar developed by the China National Rice Research Institute (CNRRI), whereas XZX-6 is a cold-tolerant cultivar developed by the Hunan Yuanjiang Agricultural Research Institute in collaboration with CNRRI [66]. LLY-7108 is a cold-tolerant hybrid cultivar, while LLY-32 is a cold-sensitive hybrid cultivar developed by the Hunan Yahua Seed Industry Research Institute. The experiment was carried out at Huazhong Agricultural University, Wuhan, China (114.37° E, 30.48° N). Plastic pots measuring 15.0 cm in height, 25.0 cm in length, and 23.0 cm in width were each filled with 10 kg of soil. Fertilizers were applied at rates of 2.5 g N, 1.54 g P, and 1.87 g K per pot. The soil was collected from the 0–20 cm surface layer of a rice paddy field and had the following physicochemical properties: pH 7.1, organic matter 6.7 g kg^−1^, Olsen-P 6.27 mg kg^−1^, exchangeable K 129 mg kg^−1^, and total N 0.63%. Sowing was carried out on 8 April 2024 by placing five seeds per pot. The crop completed a 122-day growth cycle and was harvested on 8 August 2024. Prior to CS application, seedlings were transferred to a programmable growth chamber for controlled temperature treatment. Under optimal growth conditions (pre-stress and recovery periods), the growth chamber was maintained at 60% relative humidity, a 12 h light regime at 28 °C, and a 12 h dark regime at 20 °C. For the CS treatment, the temperature was reduced to 14 °C during the light period and 10 °C during the dark period for 5 consecutive days, while relative humidity and photoperiod remained unchanged. During the recovery period, plants were returned to the natural field conditions outdoors for 7 days before physiological and growth-related measurements were taken.

### 4.2. Experimental Design and Treatments

Rice (*Oryza sativa* L.) seeds were surface-sterilized by immersion in 20% (*v*/*v*) sodium hypochlorite (NaClO) solution and subsequently soaked in ultrapure water at 30 °C for 48 h to promote germination. Five seeds were sown per pot and grown under controlled conditions in a growth chamber with 60% relative humidity, a 12 h light regime at 28 °C, and a 12 h dark regime at 20 °C. Nanoparticle treatments, including ZnO (100 mg L^−1^), Fe_2_O_3_ (50 mg L^−1^), TiO_2_ (50 mg L^−1^), and CeO_2_ (75 mg L^−1^) [39], were applied via foliar spray. For the cold stress treatment, rice seedlings 14, 21, and 28 days old were subjected to low-temperature conditions of 14 °C during the light period and 10 °C during the dark period for 5 days. Prior to stress application, the experiment was maintained in outdoor conditions, after which the pots were transferred to a growth chamber for cold treatment. Each treatment included eight replicates. Three biological replicates were randomly selected for the determination of agronomic growth parameters, two biological replicates were used for physiological and biochemical analyses, and the remaining three biological replicates were maintained until maturity for the assessment of yield and yield components. After cold exposure, the seedlings were moved back to optimal growth conditions and allowed to recover for 7 days before physiological and growth-related measurements.

### 4.3. Meteorological Data

The meteorological data were collected to characterize the environmental conditions during the experimental period, particularly during the pre-stress growth phase and post-stress recovery period when plants were maintained under natural field conditions. This information provides context for interpreting plant responses and ensures reproducibility across different growing seasons and locations. Data was collected using a data logger (CR800). Campbell Scientific Inc., Logan, UT, USA controls and collects data, recording meteorological data such as daily maximum and minimum temperatures, rainfall, and daily average radiation during the rice growing season.

### 4.4. Agronomic Characteristics

The agronomic data were collected from three pot replicates, with measurements taken from three seedlings within those replicates. After washing and drying, the seedlings’ height, fresh weight, and dry weight were recorded. Dry weight of the aboveground biomass was determined after oven-drying at 80 °C until a constant weight was achieved.

### 4.5. Chlorophyll Content Determination

Chlorophyll concentration in rice leaves was measured according to the method of Arnon [67]. Approximately 0.5 g of freshly harvested rice leaves were homogenized with 10 mL of 80% (*v*/*v*) acetone. After a 2 h period of light deprivation, the samples were centrifuged at 12,000 rpm for 10 min. Absorbance was measured at wavelengths of 663 nm and 645 nm using a spectrophotometer (Nano Quant, infinite M200, Tecan, Männedorf, Switzerland).

### 4.6. Malondialdehyde (MDA) Content Measurement

The malondialdehyde (MDA) content was quantified using the method described by Heath and Packer [68]. Approximately 0.5 g of fresh leaf tissue was homogenized in 10 mL of ice-cold 1/5 M trichloroacetic acid. The mixture was centrifuged at 12,000× *g* for 20 min. Then, 2 mL of the supernatant was mixed with an equal volume of thiobarbituric acid and incubated at 95 °C for 30 min. After cooling, the sample was centrifuged at 12,000× *g* for 30 min, and absorbance was measured at 450, 532, and 600 nm using a spectrophotometer (Nano Quant, infinite M200, Tecan, Switzerland). The MDA concentration was calculated using the following formula:

MDA (μmol/g FW) = [6.45 × (A_532_ − A_600_) − 0.56 × A_450_]/(ε × W)

where

A_532_, A_600_ and A_450_ = absorbance values at 450, 532, and 600 nm, respectively
6.45 and 0.56 = extinction coefficients for MDA
ε = extinction coefficient of MDA (155 mM^−1^ cm^−1^)
W = fresh weight of sample (g)

### 4.7. Free Proline (Pro) Content Measurement

Free proline content was measured following the method described by He et al. [69]. Approximately 0.1 g of rice leaves were ground in 3% (*v*/*v*) sulfosalicylic acid. After centrifugation at 12,000× *g* for 15 min, 2 mL of the supernatant was mixed with an equal volume of glacial acetic acid and acidic ninhydrin. The mixture was incubated at 95 °C for 40 min, then cooled on ice. Toluene (4 mL) was added to extract the proline, and the absorbance was measured at 520 nm using a spectrophotometer (Nano Quant, infinite M200, Tecan, Switzerland). A standard curve was used to determine proline concentration.

### 4.8. Antioxidant Enzyme Activity

Antioxidant enzyme activities were determined by grinding 0.5 g of fresh leaf tissue in 1 mL of cold phosphate buffer (50 mM, pH 7.8). The homogenate was centrifuged at 12,000× *g* for 20 min at 4 °C. Superoxide dismutase (SOD, EC 1.15.1) activity was measured as described by [70], peroxidase (POD, EC 1.11.1) activity was quantified using the method of [71], and catalase (CAT, EC 1.11.1.6) activity was measured following the protocol outlined by [72], using a spectrophotometer (Nano Quant, infinite M200, Tecan, Switzerland).

### 4.9. Yield and Yield Component

Once the rice plants reached maturity, yield and yield components, including grain yield per plant (GY), thousand grain weight (TGW), grain number per panicle (GN), grain filling percentage, and tillers per plant, were evaluated. For each treatment, three independent pots (biological replicates) were maintained until maturity. Each pot contained three rice plants, and yield-related traits were measured from the plants within each pot. The mean value of the three plants in each pot was calculated and used as a single biological replicate for statistical analysis, with the pot considered the experimental unit.

### 4.10. Statistical Analysis

In this study, we used Statistix 8.1 to derive ANOVAs to test the effects of chilling treatment phases, cultivars and nanoparticles application on rice growth and yield. We used Tukey’s HSD test for the multiple comparisons at the significance level of *p* < 0.05. We identified the significant main effect and corresponding interactions. Tukey’s test was used to compare means at a 5% significance level.

## 5. Conclusions and Future Perspective

**Figure 7:**
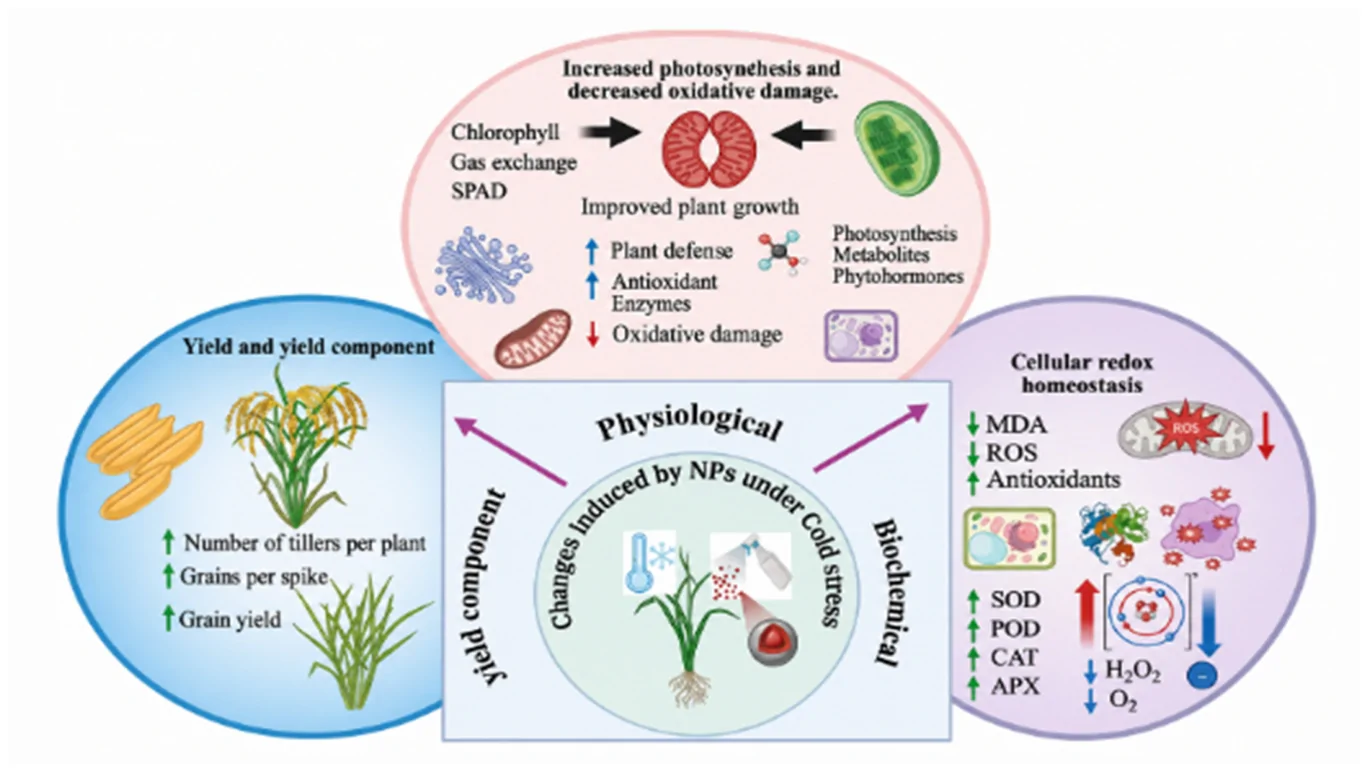
Proposed schematic model illustrating rice responses to chilling stress (14 °C/10 °C day/night). Abbreviations: H_2_O_2_, hydrogen peroxide; MDA, malondialdehyde; POD, peroxidase; SOD, superoxide dismutase; CAT, catalase; ROS, reactive oxygen species.

In conclusion, this study highlights the role of different nanoparticles (ZnO, Fe_2_O_3_, TiO_2_ and CeO_2_) in CS resilience of rice using different cultivars, i.e., cold tolerant and cold sensitive (hybrid and conventional). This provides an overview of different morphological, physiological and yield changes between different treatments under CS (Figure 7). The results of this study indicate that foliar application of ZnO, Fe_2_O_3_, TiO_2_ and CeO_2_ nanoparticles can effectively alleviate the adverse effects of cold stress (CS) in rice plants. These nanoparticles improved the plants’ ability to maintain photosynthetic pigments, reduced oxidative stress, and enhanced antioxidant enzyme activities, thereby strengthening overall stress resilience. In addition, foliar application of nanoparticles improved rice yield and yield-related components under cold stress conditions. Among the tested nanoparticles, Fe_2_O_3_ at 50 mg L^−1^ showed the highest effectiveness in mitigating cold stress damage in rice. These findings suggest that foliar application of Fe_2_O_3_ nanoparticles has the potential to mitigate the negative effects of cold stress under controlled conditions. However, further field studies are needed to validate their effectiveness and assess their practical applicability in field conditions. Furthermore, genetic and molecular studies are needed to clarify the underlying mechanisms. In particular, future research should focus on validating candidate genes, hormone-regulated pathways, and their crosstalk networks associated with Fe_2_O_3_ nanoparticle application, as well as identifying specific adaptation mechanisms that contribute to enhanced cold tolerance in rice.

## Figures and Tables

**Table 1:** Morphological parameters and photosynthetic pigments influenced by nanoparticles application and cold stress at different growth stages.

Variety		Plant Height (cm)	Fresh Weight (g/Plant)	Dry Weight (g/Plant)	Chl a (Ug/g FW)	Chl b (Ug/g FW)	Variety	Plant Height (cm)	Fresh Weight (g/Plant)	Dry Weight (g/Plant)	Chl a (Ug/g FW)	Chl b (Ug/g FW)
LLY 32	NPs						LLY 7108					
	CK	40.4 a	15.0 a	3.7 a	205.8 a	177.7 a		40.9 a	15.6 a	3.9 a	208.2 a	191.2 a
	CS	24.9 d	5.9 e	1.8 d	93.8 e	87.2 d		29.2 d	7.8 d	2.1 d	123.9 d	111.4 d
	ZnO	32.4 bc	9.1 bc	2.7 bc	155.2 bc	135.8 b		37.0 ab	10.4 c	3.2 bc	191.0 ab	174.5 b
	Fe_2_O_3_	34.1 b	10.1 b	3.0 ab	171.3 b	158.0 a		38.3 ab	13.1 b	3.8 ab	205.0 a	182.9 ab
	TiO_2_	28.4 cd	6.8 de	2.2 cd	118.6 d	99.3 cd		32.5 cd	9.0 cd	2.4 d	149.0 c	134.8 c
	CeO_2_	30.1 bc	8.2 cd	2.5 bc	138.0 c	112.7 c		34.2 bc	9.4 cd	2.7 cd	170.3 bc	146.6 c
	Stage(S)											
	Stage-I	21.2 c	6.6 c	1.0 c	142.0 a	114.1 b		24.0 c	7.9 c	1.2 c	166.8 b	143.0 b
	Stage-II	30.2 b	8.0 b	3.2 b	148.8 a	131.6 a		33.2 b	9.8 b	3.6 b	173.5 ab	160.2 a
	Stage-III	40.7 a	12.9 a	3.8 a	150.6 a	139.6 a		48.8 a	14.9 a	4.4 a	183.4 a	167.5 a
	Average	31.7 C	9.2 B	2.7 B	147.1 C	128.4 C		35.3 A	10.9 A	3.0 A	174.6 A	156.9 A
	ANOVA											
	NPs	*	*	*	*	*		*	*	*	*	*
	S	*	*	*	*	*		*	*	*	*	*
	NPs*S	ns	ns	*	ns	ns		ns	ns	*	ns	ns
XZX6	NPs						ZJZ 17					
	CK	39.5 a	15.3 a	3.9 a	205.2 a	188.7 a		40.1 a	15.0 a	3.6 a	205.3 a	189.4 a
	CS	27.5 d	7.0 e	2.0 d	120.3 d	108.3 d		23.0 e	5.4 e	1.7 c	91.9 d	81.3 e
	ZnO	35.2 abc	10.6 bc	3.0 bc	179.6 bc	153.3 b		28.9 bc	9.1 bc	2.6 b	152.3 bc	129.8 bc
	Fe_2_O_3_	36.9 ab	12.1 b	3.4 ab	194.2 ab	179.3 a		31.7 b	10.7 b	2.7 b	165.5 b	151.1 b
	TiO_2_	31.0 cd	8.4 de	2.5 cd	135.5 d	121.7 cd		24.8 de	6.9 d	1.9 c	140.6 bc	96.7 de
	CeO_2_	33.3 bc	9.2 cd	2.7 bcd	160.5 c	136.8 bc		27.4 cd	7.5 cd	2.5 b	129.6 c	116.2 cd
	Stage(S)											
	Stage-I	22.8 c	7.4 c	1.0 c	156.0 b	129.6 b		19.9 c	6.3 c	0.8 c	140.0 b	118.6 b
	Stage-II	32.7 b	9.1 b	3.6 b	162.9 b	153.8 a		28.9 b	8.1 b	3.0 b	146.4 ab	133.1 a
	Stage-III	46.2 a	14.8 a	4.2 a	178.8 a	160.6 a		39.2 a	12.9 a	3.7 a	156.4 a	130.5 ab
	Average	33.9 B	10.5 A	2.9 A	165.9 B	148.0 B		29.3 D	9.1 B	2.5 B	147.5 C	127.4 C
	ANOVA											
	NPs	*	*	*	*	*		*	*	*	*	*
	S	*	*	*	*	*		*	*	*	*	*
	NPs*S	ns	ns	ns	ns	ns		ns	ns	*	ns	ns

Numbers followed by different letters within the column are significantly different at *p* < 0.05 according to the Tukey test, “ns” means non-significant and “*” means significant.

**Table 2:** Yield and yield components of rice at different growth stages influenced by nanoparticles application and cold stress at different growth stages.

Factors	Spikeletpanicle^−1^ (NO.)	Tiller per Plant (NO.)	Thousand Grain Weight (g)	Grain Filling Percentage (%)	Grain Yield (g/Plant)
NPs					
CK	184.5 a	19.6 a	23.3	78.99 ab	66.2 a
CS	127.2 e	10.5 e	23.5	81.3 a	25.3 f
ZnO	152.5 c	13.6 c	23.8	79.9 ab	39.3 c
Fe_2_O_3_	163.7 b	15.3 b	23.8	80.9 a	48.2 b
TiO_2_	139.6 d	11.6 de	23.4	78.9 ab	30.1 e
CeO_2_	145.7 cd	12.8 cd	24.1	78.2 b	34.8 d
CV	7.02	1.17	ns	2.46	3.91
Cultivars					
LLY-7108	160.0 a	14.5 a	23.8	79.4	44.2 a
LLY-32	149.7 b	14.0 a	23.7	79.7	40.5 b
XZX-6	155.1 a	14.0 a	23.6	79.8	41.6 ab
ZJZ-17	144.0 c	13.1 b	23.5	79.9	36.4 c
CV	5.17	0.86	ns	ns	2.88
Stages					
14th day	143.8 c	12.5 c	23.9	79.8 ab	35.4 c
21st day	152.7 b	13.7 b	23.6	80.4 a	40.4 b
28th day	160.0 a	15.4 a	23.5	78.8 b	46.1 a
CV	4.09	0.68	ns	1.44	2.27
Interactions					
NPs*C	ns	ns	ns	ns	ns
C*S	ns	ns	ns	ns	ns
NPs*S	ns	*	ns	ns	*
NPs*C*S	ns	ns	ns	ns	ns

Numbers followed by different letters within the column are significantly different at *p* < 0.05 according to the Tukey test, “ns” means non-significant and “*” means significant.

**Table 3:** Characteristics of rice cultivars used in the experiment.

Cultivar	Type	Cold Tolerance	Development Institution	Key Characteristics
ZJZ-17	Conventional (indica)	Cold-sensitive	China National Rice Research Institute (CNRRI)	Early maturing, susceptible to chilling injury
XZX-6	Conventional (indica)	Cold-tolerant	Hunan Yuanjiang Agricultural Research Institute and CNRRI	Early maturing, stable performance under low temperature
LLY-7108	Hybrid (indica)	Cold-tolerant	Hunan Yahua Seed Industry Research Institute	High yield potential, strong stress tolerance
LLY-32	Hybrid (indica)	Cold-sensitive	Hunan Yahua Seed Industry Research Institute	High yield but susceptible to cold stress

## Data Availability

The datasets collected and/or analyzed in the present study are available from the corresponding authors upon reasonable request.

## Supplementary Materials

The following supporting information can be downloaded at: https://www.mdpi.com/article/10.3390/plants15172595/s1, Table S1: Relative effectiveness of each nanoparticle treatment for each cultivar; Figure S1: The relationships of plant height (cm) fresh weight (g/plant) and dry weight (g/plant) with Reactive oxygen species and membrane damage under cold stress at three different stages among four cultivars; Figure S2: The relationships of plant height (cm), fresh weight (g/plant) and dry weight with antioxidant enzymatic activity under cold stress at three different stages among four cultivars.

## References

[1] Birla D.S., Malik K., Sainger M., Chaudhary D., Jaiwal R., Jaiwal P.K. (2017). Progress and challenges in improving the nutritional quality of rice (*Oryza sativa* L.). Crit. Rev. Food Sci. Nutr..

[2] Abeysekara I., Rathnayake I. (2024). Global Trends in Rice Production, Consumption and Trade. SSRN Electron. J..

[3] Zhang T., Zhao X., Wang W., Pan Y., Huang L., Liu X., Zong Y., Zhu L., Yang D., Fu B. (2012). Comparative transcriptome profiling of chilling stress responsiveness in two contrasting rice genotypes. PLoS ONE.

[4] Mao D., Xin Y., Tan Y., Hu X., Bai J., Liu Z.-Y., Yu Y., Li L., Peng C., Fan T. (2019). Natural variation in the HAN1 gene confers chilling tolerance in rice and allowed adaptation to a temperate climate. Proc. Natl. Acad. Sci. USA.

[5] Janick J. (2012). Plant Breeding Reviews.

[6] Guo E., Wang L., Jiang S., Xiang H., Shi Y., Chen X., Cheng X., Wang X., Zhang T., Wang L. (2022). Impacts of chilling at the tillering phases on rice growth and grain yield in Northeast China. J. Agron. Crop Sci..

[7] Ullah S., Matin M.N., Yin C., Mas-Ud A., Khan A., Islam S., Irfanullah, Haq I.U. (2026). Phytohormone-Mediated Regulation of Plant Cold Stress Tolerance: Signaling, Hormonal Crosstalk, and Translational Perspectives. Int. J. Mol. Sci..

[8] Shimono H., Ishii A., Kanda E., Suto M., Nagano K. (2011). Genotypic variation in rice cold tolerance responses during reproductive growth as a function of water temperature during vegetative growth. Crop Sci..

[9] Baruah A.R., Ishigo-Oka N., Adachi M., Oguma Y., Tokizono Y., Onishi K., Sano Y. (2009). Cold tolerance at the early growth stage in wild and cultivated rice. Euphytica.

[10] Yang X., Liu Z., Chen F. (2011). The possible effect of climate warming on northern limits of cropping system and crop yield in China. Agric. Sci. China.

[11] Yang X., Lin E., Ma S., Ju H., Guo L., Xiong W., Li Y. (2007). Adaptation of agriculture to warming in Northeast China. Clim. Change.

[12] Chen H., Sun J. (2015). Changes in climate extreme events in China associated with warming. Int. J. Climatol..

[13] Liu X., Zhang Z., Shuai J., Wang P., Shi W., Tao F., Chen Y. (2013). Impact of chilling injury and global warming on rice yield in Heilongjiang Province. J. Geogr. Sci..

[14] Jiang L., Han J., Cui H., Chu Z., Li S., Zhang Y., Ji Y., Wang Q., Li X., Wang P. (2024). Effect of Climate Change on Identification of Delayed Chilling Damage of Rice in China’s Cold Region. Agriculture.

[15] Shi Y., Guo E., Cheng X., Wang L., Jiang S., Yang X., Ma H., Zhang T., Li T., Yang X. (2022). Effects of chilling at different growth stages on rice photosynthesis, plant growth, and yield. Environ. Exp. Bot..

[16] White J.C., Gardea-Torresdey J. (2018). Achieving food security through the very small. Nat. Nanotechnol..

[17] Pulizzi F. (2019). Nano Future crops. Nat. Nanotechnol..

[18] Zhao L., Lu L., Wang A., Zhang H., Huang M., Wu H., Xing B., Wang Z., Ji R. (2020). Nano-biotechnology in agriculture: Use of nanomaterials to promote plant growth and stress tolerance. J. Agric. Food Chem..

[19] Wu H., Ge Y. (2019). Excessive application of fertilizer, agricultural non-point source pollution, and farmers’ policy choice. Sustainability.

[20] Rassaei F. (2025). The Role of Ferric oxide in Reducing Carbon Dioxide Emissions from Rice cultivation: A Pot Greenhouse Experiment. Water Air Soil Pollut..

[21] Sharifi Rad J., Karimi J., Mohsenzadeh S., Rad M.S., Moradgohli J. (2014). Evaluating SiO_2_ nanoparticles effects on developmental characteristic and photosynthetic pigment contents of *Zea mays* L. Bull. Environ. Pharm. Life Sci..

[22] Wang C., Ji Y., Cao X., Yue L., Chen F., Li J., Yang H., Wang Z., Xing B. (2022). Carbon dots improve nitrogen bioavailability to promote the growth and nutritional quality of soybeans under drought stress. ACS Nano.

[23] Feng Y., Kreslavski V.D., Shmarev A.N., Ivanov A.A., Zharmukhamedov S.K., Kosobryukhov A., Yu M., Allakhverdiev S.I., Shabala S. (2022). Effects of iron oxide nanoparticles (Fe_3_O_4_) on growth, photosynthesis, antioxidant activity and distribution of mineral elements in wheat (*Triticum aestivum*) plants. Plants.

[24] Lord M.S., Berret J.F., Singh S., Vinu A., Karakoti A.S. (2021). Redox active cerium oxide nanoparticles: Current status and burning issues. Small.

[25] Nie L., Zhou G., Yin Y., Guo X., He A., Li S., Wu G., Zhang R., Zeng Y., Chen H. (2026). Foliar Titanium Dioxide Nanoparticles Enhance Rice Yield by Improving Photosynthesis, Ion Balance, and Antioxidant Defense Under Salt Stress. Plants.

[26] Song Y., Jiang M., Zhang H., Li R. (2021). Zinc oxide nanoparticles alleviate chilling stress in rice (*Oryza sativa* L.) by regulating antioxidative system and chilling response transcription factors. Molecules.

[27] Yang G., Xia Y., Ren W. (2021). Glutamine metabolism in Th17/Treg cell fate: Applications in Th17 cell-associated diseases. Sci. China Life Sci..

[28] Pandey S.N. (2018). Biomolecular functions of micronutrients toward abiotic stress tolerance in plants. Plant Nutrients and Abiotic Stress Tolerance.

[29] Sedghi M., Hadi M., Toluie S.G. (2013). Effect of nano zinc oxide on the germination parameters of soybean seeds under drought stress. Ann. West Univ. Timis. Ser. Biol..

[30] Torabian S., Zahedi M., Khoshgoftar A.H. (2016). Effects of foliar spray of two kinds of zinc oxide on the growth and ion concentration of sunflower cultivars under salt stress. J. Plant Nutr..

[31] Jahed K.R., Saini A.K., Sherif S.M. (2023). Coping with the cold: Unveiling cryoprotectants, molecular signaling pathways, and strategies for cold stress resilience. Front. Plant Sci..

[32] Cao Y., Turk K., Bibi N., Ghafoor A., Ahmed N., Azmat M., Ahmed R., Ghani M.I., Ahanger M.A. (2025). Nanoparticles as catalysts of agricultural revolution: Enhancing crop tolerance to abiotic stress: A review. Front. Plant Sci..

[33] Wang G.J., Miao W., Wang J., Ma D., Li J., Chen W. (2013). Effects of exogenous abscisic acid on antioxidant system in weedy and cultivated rice with different chilling sensitivity under chilling stress. J. Agron. Crop Sci..

[34] Gautam K., Singh H., Sinha A. (2025). Nanotechnology in Plant Nanobionics: Mechanisms, Applications, and Future Perspectives. Adv. Biol..

[35] Newkirk G.M., de Allende P., Jinkerson R.E., Giraldo J.P. (2021). Nanotechnology approaches for chloroplast biotechnology advancements. Front. Plant Sci..

[36] Ali L., Manzoor N., Masood H.A., Abbas A. (2024). Nanotechnology-Enabled Approaches to Mitigating Abiotic Stresses in Agricultural Crops. Molecular Dynamics of Plant Stress and Its Management.

[37] Al-Khayri J.M., Rashmi R., Ulhas R.S., Sudheer W.N., Banadka A., Nagella P., Aldaej M.I., Rezk A.A.-S., Shehata W.F., Almaghasla M.I. (2023). The role of nanoparticles in response of plants to abiotic stress at physiological, biochemical, and molecular levels. Plants.

[38] Wang L., Ning C., Pan T., Cai K. (2022). Role of silica nanoparticles in abiotic and biotic stress tolerance in plants: A review. Int. J. Mol. Sci..

[39] Ullah S., Ikram M., Xiao J., Khan A., Din I., Huang J. (2024). Influence of foliar application of nanoparticles on low temperature resistance of Rice seedlings. Plants.

[40] Zhu J., Zhang K.-X., Wang W.-S., Gong W., Liu W.-C., Chen H.-G., Xu H.-H., Lu Y.-T. (2015). Low temperature inhibits root growth by reducing auxin accumulation via ARR1/12. Plant Cell Physiol..

[41] Kroh G.E., Pilon M. (2020). Regulation of iron homeostasis and use in chloroplasts. Int. J. Mol. Sci..

[42] Arnon D.I. (1965). Ferredoxin and Photosynthesis: An iron-containing protein is a key factor in energy transfer during photosynthesis. Science.

[43] Raven J.A., Evans M.C., Korb R.E. (1999). The role of trace metals in photosynthetic electron transport in O_2_-evolving organisms. Photosynth. Res..

[44] Malone L.A., Proctor M.S., Hitchcock A., Hunter C.N., Johnson M.P. (2021). Cytochrome b6f–Orchestrator of photosynthetic electron transfer. Biochim. Biophys. Acta (BBA)-Bioenerg..

[45] Caccamo A. (2024). The Role of Ascorbate Peroxidase 2 and Protein Cysteine Modifications in the Green Alga Chlamydomonas Reinhardtii. Ph.D. Thesis.

[46] Wang H., Zhong L., Fu X., Huang S., Zhao D., He H., Chen X. (2023). Physiological analysis reveals the mechanism of accelerated growth recovery for rice seedlings by nitrogen application after low temperature stress. Front. Plant Sci..

[47] Sato Y., Masuta Y., Saito K., Murayama S., Ozawa K. (2011). Enhanced chilling tolerance at the booting stage in rice by transgenic overexpression of the ascorbate peroxidase gene, OsAPXa. Plant Cell Rep..

[48] Xie G., Kato H., Imai R. (2012). Biochemical identification of the OsMKK6–OsMPK3 signalling pathway for chilling stress tolerance in rice. Biochem. J..

[49] Manasa L., Panigrahy M., Panigrahi K.C.S. (2022). Overview of cold stress regulation in plants. Bot. Rev..

[50] Huang J., Sun S., Xu D., Lan H., Sun H., Wang Z., Bao Y., Wang J., Tang H., Zhang H. (2012). A TFIIIA-type zinc finger protein confers multiple abiotic stress tolerances in transgenic rice (*Oryza sativa* L.). Plant Mol. Biol..

[51] Khalid M.F., Khan R.I., Jawaid M.Z., Shafqat W., Hussain S., Ahmed T., Rizwan M., Ercisli S., Pop O.L., Marc R.A. (2022). Nanoparticles: The plant saviour under abiotic stresses. Nanomaterials.

[52] Hossen M.S., Karim M.F., Fujita M., Bhuyan M.H.M.B., Nahar K., Masud A.A.C., Mahmud J.A., Hasanuzzaman M. (2022). Comparative physiology of indica and japonica rice under salinity and drought stress: An intrinsic study on osmotic adjustment, oxidative stress, antioxidant defense and methylglyoxal detoxification. Stresses.

[53] Lu X., Zhou Y., Fan F., Peng J., Zhang J. (2020). Coordination of light, circadian clock with temperature: The potential mechanisms regulating chilling tolerance in rice. J. Integr. Plant Biol..

[54] Sohag A.A.M., Tahjib-Ul-Arif M., Afrin S., Khan K., Hannan A., Skalicky M., Mortuza G., Brestic M., Hossain M.A., Murata Y. (2020). Insights into nitric oxide-mediated water balance, antioxidant defence and mineral homeostasis in rice (*Oryza sativa* L.) under chilling stress. Nitric Oxide.

[55] Xiao M., Li Z., Zhu L., Wang J., Zhang B., Zheng F., Zhao B., Zhang H., Wang Y., Zhang Z. (2021). The multiple roles of ascorbate in the abiotic stress response of plants: Antioxidant, cofactor, and regulator. Front. Plant Sci..

[56] Zhao Y., Han Q., Ding C., Huang Y., Liao J., Chen T., Feng S., Zhou L., Zhang Z., Chen Y. (2020). Effect of low temperature on chlorophyll biosynthesis and chloroplast biogenesis of rice seedlings during greening. Int. J. Mol. Sci..

[57] Wang W., Chen Q., Hussain S., Mei J., Dong H., Peng S., Huang J., Cui K., Nie L. (2016). Pre-sowing seed treatments in direct-seeded early rice: Consequences for emergence, seedling growth and associated metabolic events under chilling stress. Sci. Rep..

[58] Wang W., Peng S., Chen Q., Mei J., Dong H., Nie L. (2016). Effects of pre-sowing seed treatments on establishment of dry direct-seeded early rice under chilling stress. AoB Plants.

[59] Mo Z., Ashraf U., Pan S., Kanu A., Li W., Duan M., Tian H., Tang X. (2016). Exogenous application of plant growth regulators induce chilling tolerance in direct seeded super and non-super rice seedlings through modulations in morpho-physiological attributes. Cereal Res. Commun..

[60] El-Refaee Y.Z., Gharib H.S., Badawy S.A., Elrefaey E.M., El-Okkiah S.A., Okla M.K., Maridueña-Zavala M.G., AbdElgawad H., El-Tahan A.M. (2024). Mitigating cold stress in rice: A study of genotype performance and sowing time. BMC Plant Biol..

[61] Zhang C.X., Guo B.W., Tang J., Xu F.F., Xu K., Hu Y.J., Xing Z.P., Zhang H.C., Dai Q.G., Huo Z.Y. (2019). Combined effects of low temperature and weak light at grain-filling stage on rice grain quality. Acta Agron. Sin..

[62] Ding M., Xiao C., Li S., Liu J., Kong Y., Zhang C., Li H., Yang Y., Liu K., Rehman M. (2025). Effects of iron, zinc, and silicon nanoparticles on morpho-physiological growth, yield, and quality of wheat (*Triticum aestivum* L.) under drought stress. J. Hazard. Mater. Adv..

[63] Elekhtyar N.M., AAl-Huqail A. (2023). Effect of foliar application of phosphorus, zinc, and silicon nanoparticles along with mineral NPK fertilization on yield and chemical compositions of rice (*Oryza sativa* L.). Agriculture.

[64] Shukla A.K., Ladha J.K., Singh V.K., Dwivedi B.S., Balasubramanian V., Gupta R.K., Sharma S.K., Singh Y., Pathak H., Pandey P.S. (2004). Calibrating the leaf color chart for nitrogen management in different genotypes of rice and wheat in a systems perspective. Agron. J..

[65] Shrestha S., Asch F., Dusserre J., Ramanantsoanirina A., Brueck H. (2012). Climate effects on yield components as affected by genotypic responses to variable environmental conditions in upland rice systems at different altitudes. Field Crops Res..

[66] Tang S. (2019). Effects of Low Temperature and Water-Logging Stress at Bus Stage on Growth Characteristics and Yield of Early Indica Rice. Master’s Thesis.

[67] Arnon D.I. (1949). Copper enzymes in isolated chloroplasts. Polyphenoloxidase in Beta vulgaris. Plant Physiol..

[68] Heath R.L., Packer L. (1968). Photoperoxidation in isolated chloroplasts: I. Kinetics and stoichiometry of fatty acid peroxidation. Arch. Biochem. Biophys..

[69] He J., Duan Y., Hua D., Fan G., Wang L., Liu Y., Chen Z., Han L., Qu L.-J., Gong Z. (2012). DEXH box RNA helicase–mediated mitochondrial reactive oxygen species production in Arabidopsis mediates crosstalk between abscisic acid and auxin signaling. Plant Cell.

[70] Ulusu Y., Öztürk L., Elmastaş M. (2017). Antioxidant capacity and cadmium accumulation in parsley seedlings exposed to cadmium stress. Russ. J. Plant Physiol..

[71] Sharma P., Jha A.B., Dubey R.S., Pessarakli M. (2012). Reactive oxygen species, oxidative damage, and antioxidative defense mechanism in plants under stressful conditions. J. Bot..

[72] Karabal E., Yücel M., Öktem H.A. (2003). Antioxidant responses of tolerant and sensitive barley cultivars to boron toxicity. Plant Sci..

