# Supplementary material for: Influence of Nanoparticles on Morpho-Physiological, Growth and Yield Traits of Rice (Oryza sativa L.) Cultivars Under Early Seedling Cold Stress at Different Developmental Stages
Source: Plants (Basel). 2026 Aug 25;15(17):2595. doi: 10.3390/plants15172595 (PMC13567719; doi:10.3390/plants15172595)
Supplement: Supplementary file 1 [file plants-15-02595-s001.zip › plants-4460796-supplementary.pdf]

Table S1. Relative effectiveness of each nanoparticle treatment for each cultivar

Cultivar	Most Effective NP	Least Effective NP	Overall Tolerance
LLY-7108	Fe_2O_3 > ZnO > CeO_2 > TiO_2	TiO_2	High
XZX-6	Fe_2O_3 > ZnO > CeO_2 > TiO_2	TiO_2	High
LLY-32	Fe_2O_3 > ZnO > CeO_2 > TiO_2	TiO_2	Moderate
ZJZ-17	Fe_2O_3 > ZnO > CeO_2 > TiO_2	TiO_2	Low

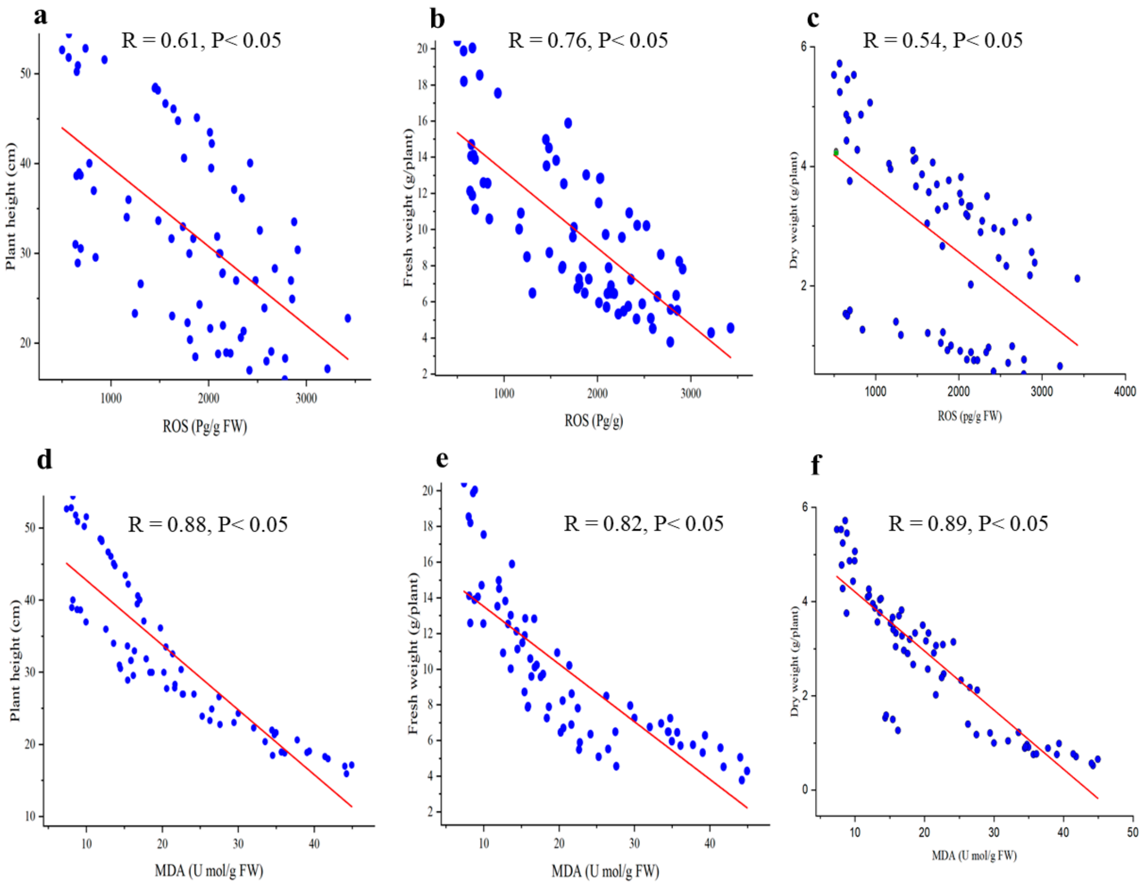

Figure S1. The relationships of plant height (cm) fresh weight (g/plant) and dry weight (g/plant) with Reactive oxygen species and membrane damage under cold stress at three different stages among four cultivars. The temperature was set at 14/10 °C for cold treatment for 5 days, while the data was recorded after 7 days of recovery.

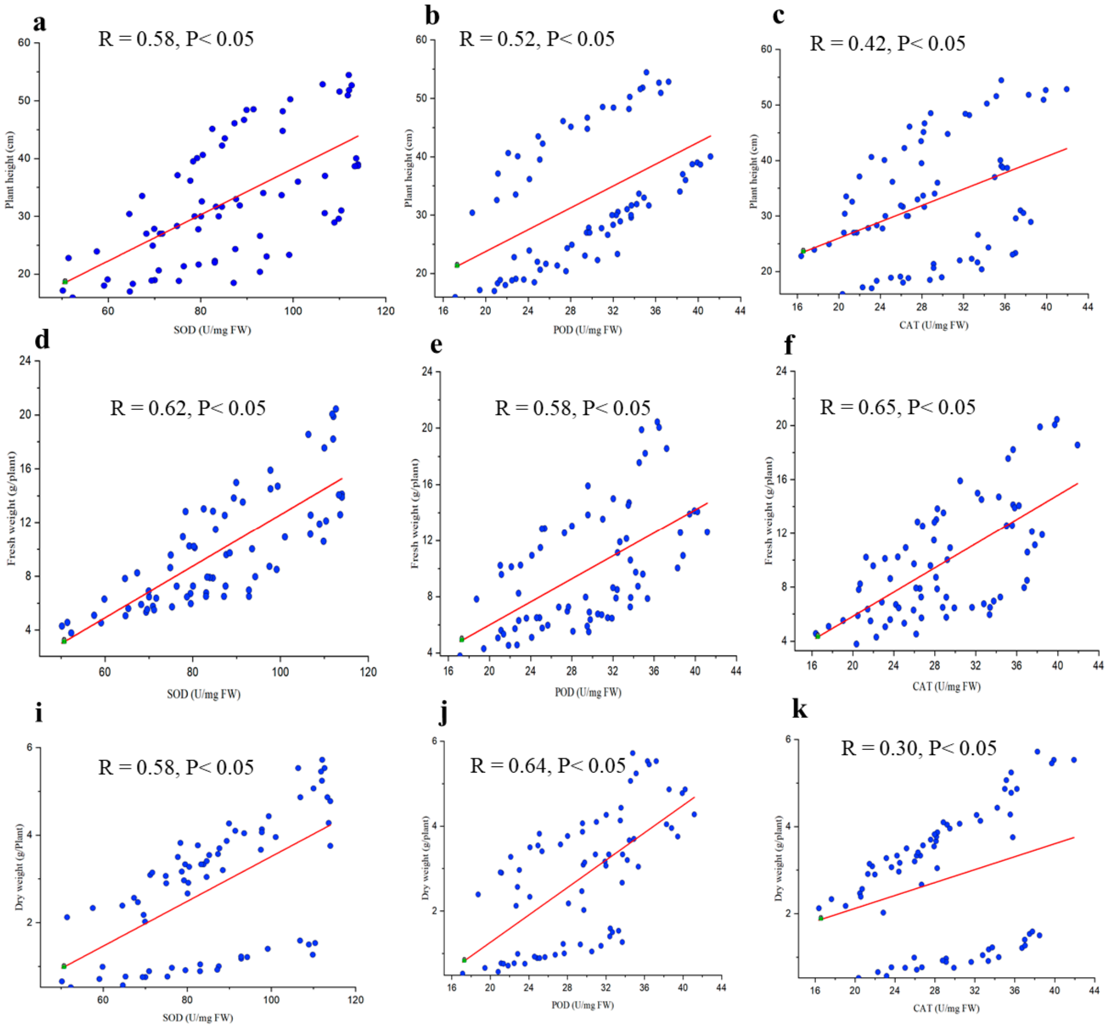

Figure S2. The relationships of plant height (cm), fresh weight (g/plant) and dry weight with antioxidant enzymatic activity under cold stress at three different stages among four cultivars. The temperature was set at 14/10 °C for cold treatment for 5 days, while the data was recorded after 7 days of recovery.
